# Multi-omics and machine learning identify FN1 and ALDH2 as diagnostic biomarkers and therapeutic targets in early and late diabetic kidney disease

**DOI:** 10.1080/0886022X.2025.2577849

**Published:** 2025-10-29

**Authors:** Jingwei Lin, Yingying Zheng, Diman Mai, Fuxiang Fang, Zhuokun Wei, Mengyu Liu, Trung Hieu Pham, Ming Li, Jiawen Zhao

**Affiliations:** a Department of Urology, The First Affiliated Hospital of Guangxi Medical University, Guangxi Medical University, Nanning, Guangxi, China; b Fangchenggang Hospital of Traditional Chinese Medicine, Guangxi University of Chinese Medicine, Fangchenggang, Guangxi, China; c Center for Genomic and Personalized Medicine, Guangxi Key Laboratory for Genomic and Personalized Medicine, Guangxi Collaborative Innovation Center for Genomic and Personalized Medicine, Guangxi Medical University, Nanning, Guangxi, China; d Department of Oncology, The First Affiliated Hospital of Guangxi Medical University, Guangxi Medical University, Nanning, Guangxi, China

**Keywords:** Diabetic kidney disease, disease progression, multi-omics integration, fibrosis, oxidative stress, precision nephrology

## Abstract

Diabetic kidney disease (DKD), the leading cause of end-stage kidney disease worldwide, demands deeper molecular characterization to improve clinical management. This study employed an integrated multi-omics approach to identify stage-specific biomarkers and molecular mechanisms distinguishing early- and late-stage DKD. Initial bulk RNA-seq analysis (thresholds: |logFC|>0.585, FDR < 0.05) revealed differentially expressed genes and enriched pathways, followed by two-sample Mendelian randomization (IVW *p* < 0.05) to pinpoint causal genes. Diagnostic modeling combined LASSO regression (10-fold cross-validation) with four machine learning algorithms (Random Forest, SVM, GLM, and XGBoost), validated in an independent cohort. Single-cell resolution analysis mapped candidate gene expression patterns, while molecular docking screened potential therapeutics. Through an integrated validation pipeline, FN1 (OR = 1.32) and ALDH2 (OR = 0.76) were established as core diagnostic biomarkers. FN1 (upregulated in mesangial cells) and ALDH2 (downregulated in proximal tubules) were validated as stage-specific biomarkers of DKD progression. FN1 expression negatively correlated with eGFR decline, while ALDH2 positively correlated. Resveratrol showed high-affinity docking to FN1 and ALDH2, suggesting dual-target therapeutic potential. These findings position FN1, ALDH2, and resveratrol as central references for DKD biomarker discovery, progression staging, and therapeutic development.

## Introduction

Diabetic kidney disease (DKD), one of the most prevalent complications of diabetes, affects 30–40% of individuals with diabetes [1]. The Global Burden of Diseases study reported that 529 million people worldwide had diabetes in 2021, with an age-standardized global prevalence of 6.1% [2]. Notably, among adults aged 20–79 years, the current diabetes prevalence reaches 10.5%, and this figure is projected to rise to 12.2% by 2045[3]. Despite recent advances in nephroprotective therapies—including renin-angiotensin system inhibitors, sodium-glucose cotransporter-2 (SGLT2) inhibitors, and non-steroidal mineralocorticoid receptor antagonists (MRAs)—these agents fail to reverse the substantial disease burden imposed by DKD [4]. Consequently, elucidating the molecular mechanisms underlying DKD progression, identifying robust biomarkers for early diagnosis and treatment, and developing novel therapeutic strategies to halt or reverse DKD are critical unmet needs.

The pathogenesis of DKD involves a complex interplay of hyperglycemia-driven mechanisms, such as inflammatory responses, oxidative stress, epithelial-mesenchymal transition (EMT), cellular senescence, and apoptosis [5,6]. Recent advances in multi-omics approaches have facilitated the discovery of novel biomarkers for DKD [7]. For instance, machine learning and single-cell RNA sequencing identified VCAN as a shared biomarker in glomerular parietal epithelial cells and proximal tubules, demonstrating diagnostic utility for estimated glomerular filtration rate (eGFR) decline and proteinuria [8]. Similarly, integrative multi-omics analyses by Li et al. revealed PTEN as a key protective gene against cellular senescence in DKD [9], while another study leveraged machine learning to pinpoint four diagnostic biomarkers (TNC, PXDN, TIMP1, and TPM1) linked to oxidative stress and inflammation [10]. Proteomic profiling further uncovered peroxisomal dysfunction and mitochondrial metabolic disturbances in both early and late-stage DKD in murine models, offering new insights into its molecular underpinnings [11]. While significant progress has been made, two fundamental challenges persist: the lack of biomarkers with combined diagnostic and therapeutic utility, and insufficient systematic characterization of molecular distinctions between early and late-stage DKD.

This study implemented an integrated multi-omics strategy to delineate stage-specific molecular signatures in DKD. Bulk RNA sequencing data encompassing early-stage DKD, advanced DKD, and healthy controls were systematically analyzed through differential expression profiling and functional enrichment approaches, with focused investigation of critical pathological pathways including oxidative stress, inflammatory responses, cellular senescence, EMT, apoptosis, and autophagy. Leveraging multiple machine learning algorithms augmented by Mendelian randomization (MR), we established a diagnostic model demonstrating robust performance across both training and validation cohorts. Molecular docking simulations further identified resveratrol as a potential therapeutic agent through its predicted interactions with key biomarkers. At single-cell resolution, we further analyzed candidate biomarkers at the cellular level and performed pathway enrichment analyses of key DKD pathological processes, revealing molecular heterogeneity between early and late disease stages. By implementing this comprehensive integrative framework, we aimed to rigorously validate and establish stage-specific molecular signatures and biomarkers with high diagnostic and therapeutic potential, thereby providing a more precise tool for DKD staging and targeted intervention.

## Materials and methods

### Data collection and preprocessing

8 DKD-related Gene Expression Omnibus (GEO) datasets were analyzed, including 3 single-cell RNA-seq (GSE131882, GSE209781, GSE266146) and 5 bulk RNA-seq datasets (GSE96804, GSE104948, GSE104954, GSE142025, GSE30529). GSE96804 served as the training set, with GSE104954/GSE104948 as the independent validation set for biomarker confirmation. GSE142025 and GSE30529 enabled a systematic comparison of molecular features between early- and late-stage DKD. 3 independent single-cell RNA sequencing datasets encompassing both kidney tissue and urinary samples. The kidney tissue single-cell RNA-seq datasets included GSE131882 and GSE209781, while the urinary sediment single-cell RNA-seq dataset comprised GSE266146. The detailed characteristics of these renal tissue sequencing samples are comprehensively described in the original publications associated with each dataset (https://www.ncbi.nlm.nih.gov/geo/, accessed January 8, 2025). Genes mapped to multiple probes were calculated by their average values. Detailed descriptions of the GEO datasets are provided in Table S1.

The exposure genome-wide association study (GWAS) data were obtained from the OpenGWAS database (https://gwas.mrcieu.ac.uk/, accessed January 15, 2025). We selected genome-wide significant expression quantitative trait loci (eQTL)-related SNPs (*p* < 5 × 10^−8^) and performed linkage disequilibrium (LD) clumping (*r*^2^ < 0.001 within 10,000 kb windows), ultimately retaining 26,152 independent SNPs associated with 5,430 distinct genes for subsequent MR analysis. The DM_NEPHROPATHY_EXMOR dataset (64,663 individuals) was obtained from FinnGen Release R12 (https://www.finngen.fi/en/access_results, accessed February 15, 2025), while the GCST90435706 dataset (388,955 individuals) was acquired from the GWAS Catalog (https://www.ebi.ac.uk/gwas/, accessed February 15, 2025) as outcome GWAS data for DKD. Both exposure and outcome GWAS datasets were restricted to European-ancestry populations to minimize population stratification. As all analyses used de-identified, publicly available data, no additional ethical approval or participant consent was required.

Gene sets were acquired from multiple established sources. Hallmark and Kyoto Encyclopedia of Genes and Genomes (KEGG) pathways relevant to inflammatory response, epithelial-mesenchymal transition (EMT), and apoptosis were retrieved from the Molecular Signatures Database (MSigDB) [12]. Autophagy-related genes were sourced from the Human Autophagy Database, while gene sets associated with cellular senescence [13] and oxidative stress [14] were systematically curated from published literature. The complete gene lists for all pathways are available in Table S2.

### Differential expression analysis and functional enrichment

Transcriptomic profiling data of DKD were obtained from the GSE142025 dataset. Differentially expression genes (DEGs) analysis was performed using the limma package (v 3.64.1), applying a significance threshold of adjusted *p* < 0.05 with |logFC| > 0.585. The Benjamini-Hochberg method was implemented to control the false discovery rate (FDR).

For functional characterization, Hallmark pathway enrichment analyses were performed using clusterProfiler (v 4.16.0) with org.Hs.eg.db annotations (v 3.21.0), and results were visualized *via* enrichplot (v 1.28.2). Gene set enrichment analysis (GSEA) used Hallmark gene sets from MSigDB (adjusted *p* < 0.05), with the top five enriched pathways visualized. To further characterize molecular differences between DKD stages, single-sample GSEA (ssGSEA) was implemented using GSVA (v 2.2.0), focusing on oxidative stress, inflammatory response, EMT, cellular senescence, apoptosis, and autophagy gene sets. All findings were validated in the independent cohort GSE30529 using identical thresholds.

### Causal gene identification *via* mendelian randomization

Our analysis adhered to three core MR assumptions: (1) the relevance assumption, which posits that the SNP is strongly associated with the exposure; (2) the exclusion restriction assumption, which requires that the SNP is not associated with the outcome; and (3) the independence assumption, which necessitates that the SNP is not correlated with confounding factors [15]. To ensure the strength of our genetic instruments and mitigate weak instrument bias, we rigorously quantified the instrumental variable strength for each SNP. The proportion of variance explained (*R*^2^) in the exposure by each individual SNP was calculated using the formula: (2×EAF×(1−EAF)×beta^2)/[(2×EAF×(1−EAF)×β2)+(2×EAF×(1−EAF)×N×SE(β)2), where EAF is the effect allele frequency, β and SE are the effect size and its standard error from the eQTL study, and N is the eQTL sample size. The strength of each instrument was then assessed using the F-statistic, derived from the *R*^2^ value: *F* = [*R*^2^ × (*N* − 1)]/(1 − *R*^2^). An F-statistic greater than 10 is a widely accepted threshold indicating a sufficiently strong instrument that minimizes bias [16]. Accordingly, all SNPs with an F-statistic of less than 10 were excluded from subsequent MR analyses. Besides, potential confounding factors were addressed using phenoscanner screening. Additional quality control included removal of SNPs showing association with DKD (*p* < 5 × 10^−6^) and palindromic SNPs during harmonization. We analyzed summary-level data from two independent cohorts: the DM_NEPHROPATHY_EXMOR dataset (Finnish population, *N* = 64,663) and the GCST90435706 dataset (British population, *N* = 388,955), with no sample overlap confirmed by distinct recruitment populations and genotyping platforms.

Primary analysis used inverse variance-weighted (IVW) estimation (TwoSampleMR package v0.6.16) [17], with a significance threshold of *p* < 0.05. We complemented this with MR-Egger regression and weighted median approaches. Sensitivity analyses included Cochran’s *Q* test and MR-Egger intercept test to evaluate heterogeneity and horizontal pleiotropy [18,19] (*p* > 0.05 considered nonsignificant), steiger directionality analysis to determine the directionality of each exposure, MR-PRESSO analysis for outlier detection [20] and leave-one-out analysis to assess result stability through iterative SNP exclusion. Complementary differential expression analysis of the GSE96804 dataset using limma (v3.64.1) identified DEGs at adjusted *p* < 0.05 with |logFC| > 0.585. Integration of MR and transcriptomic data involved intersecting DKD high-expression genes (logFC > 0.585) with MR risk genes (OR > 1), and DKD low-expression genes (logFC < −0.585) with MR protective genes (OR < 1), revealing potential causal genes with concordant effects across both transcriptomic and MR analyses.

### Machine learning approaches

To develop a robust diagnostic signature for DKD, feature selection was first performed using least absolute shrinkage and selection operator (LASSO) regression with 10-fold cross-validation through the glmnet package (v 4.1.9). The optimal penalty parameter (λ) was determined by minimizing binomial deviance (λmin), retaining genes with non-zero coefficients for subsequent analysis. The diagnostic potential of these genes was then evaluated by receiver operating characteristic (ROC) analysis (pROC package, v 1.18.5), where genes exhibiting an AUC > 0.8 in both training and test datasets were selected for multivariate modeling.

The final predictive signature was constructed by integrating selected genes through four machine learning approaches: random forest (RF; randomForest package, v4.7.1.2), support vector machines (SVM) with radial basis kernel (kernlab package, v0.9.33), eXtreme Gradient Boosting (XGBoost; xgboost package, v1.7.11.1), and generalized linear model (GLM). Batch effects across datasets were corrected using the sva package (v 3.56.0) before analysis. Model performance was assessed on the independent validation cohort. Discrimination was evaluated using the area under the receiver operating characteristic curve (AUC). Calibration was visually inspected using a calibration plot. Complementing this, decision curve analysis (rmda package v1.6) was employed to evaluate the model’s clinical utility by calculating net benefit across probability thresholds [21].

### Clinical correlation analysis

Pearson correlation analysis was performed to assess associations between identified biomarker expression levels and renal function parameters in DKD patients using the Nephroseq v5 database (http://v5.nephroseq.org, accessed March 4, 2025). The primary analysis focused on the relationship with estimated eGFR calculated using the Modification of Diet in Renal Disease equation.

### Molecule docking analysis

Potential therapeutic compounds were systematically identified through the following workflow: First, clinically actionable compounds targeting the identified diagnostic biomarkers were systematically screened using the DSigDB database, which aggregates experimentally validated and computationally predicted compound-target relationships. Then, the molecular structure for resveratrol (CID:445154) was retrieved from the PubChem Compound database (http://www.ncbi.nlm.nih.gov/pccompound, accessed March 20, 2025). The three-dimensional coordinates of candidate biomarkers were downloaded from the RCSB Protein Data Bank. Hydrogen atoms were added, and all water molecules were removed during processing. Finally, molecular docking was performed using CB-Dock2[22], which automatically identifies potential binding pockets and calculates interaction stability through Vina scores (more negative values indicate stronger binding). Vina scores below −5.0 suggest significant affinity between the ligand and receptor [23], with subsequent visualization of optimal protein-ligand binding conformations.

### Single‑cell RNA‑seq analysis

Quality control was performed using Seurat (v 5.3.0) with the following exact parameters: genes detected in fewer than three cells were excluded, along with cells containing fewer than two hundred detected genes during initial filtering. Subsequent filtering removed cells with either fewer than three hundred or more than five thousand detected genes and those exceeding fifteen percent mitochondrial content. Following UMI count normalization (scale factor = 10,000), dimensionality reduction was conducted using the top thirty principal components for visualization with t-distributed Stochastic Neighbor Embedding (t-SNE) and clustering analysis. Graph-based clustering was then implemented at empirically optimized resolutions to delineate cellular subpopulations. Cell type annotation referenced canonical markers from established renal single-cell studies [24–27].

Functional enrichment analysis was performed using the AddModuleScore method to evaluate activity levels of biologically relevant pathways, including oxidative stress, inflammatory response, EMT, cellular senescence, apoptosis, and autophagy. For developmental trajectory reconstruction, the monocle package (v 2.36.0) [28–30] was implemented with its core Reversed Graph Embedding algorithm to construct pseudotime ordering. This involved dimensionality reduction followed by cell state ordering along computationally inferred trajectories. The expression dynamics of machine learning-derived candidate genes were systematically mapped across the renal cellular landscape to characterize their transitional patterns.

Intercellular communication networks were analyzed using CellChat (v 2.2.0) by systematically evaluating ligand-receptor pair expression across cell populations, identifying potential interaction patterns between distinct cell types [31]. Significantly altered cell-cell communication events were visualized to delineate interaction networks differentially active in DKD versus controls. Differential gene expression analysis between cell subtypes was performed using the FindMarkers function. Functional pathway activity was quantified through single-sample gene set enrichment analysis (ssGSEA) using the GSVA package (v 2.2.0), with 186 KEGG gene sets curated from MSigDB.

Single-cell RNA sequencing data obtained from urinary sediments of patients with early-stage and late-stage DKD (GSE266146) were processed using the standard Seurat pipeline (v5.3.0). The data were integrated based on genes shared across all samples following standardized quality control procedures. Differential gene expression analysis between early- and late-stage DKD was subsequently conducted, with the results visualized through violin plots.

### Statistical analyses

All statistical analyses were performed using R software (v 4.5.1). The Pearson correlation coefficient was used to evaluate associations between biomarker expression levels and eGFR. Group comparisons were conducted using appropriate statistical tests (t-tests, chi-square tests, Fisher’s exact test, or ANOVA) based on variable characteristics. All tests were two-sided, with statistical significance set at *p* < 0.05.

## Results

### Identification of DEGs and enrichment analysis

**Figure 1.**
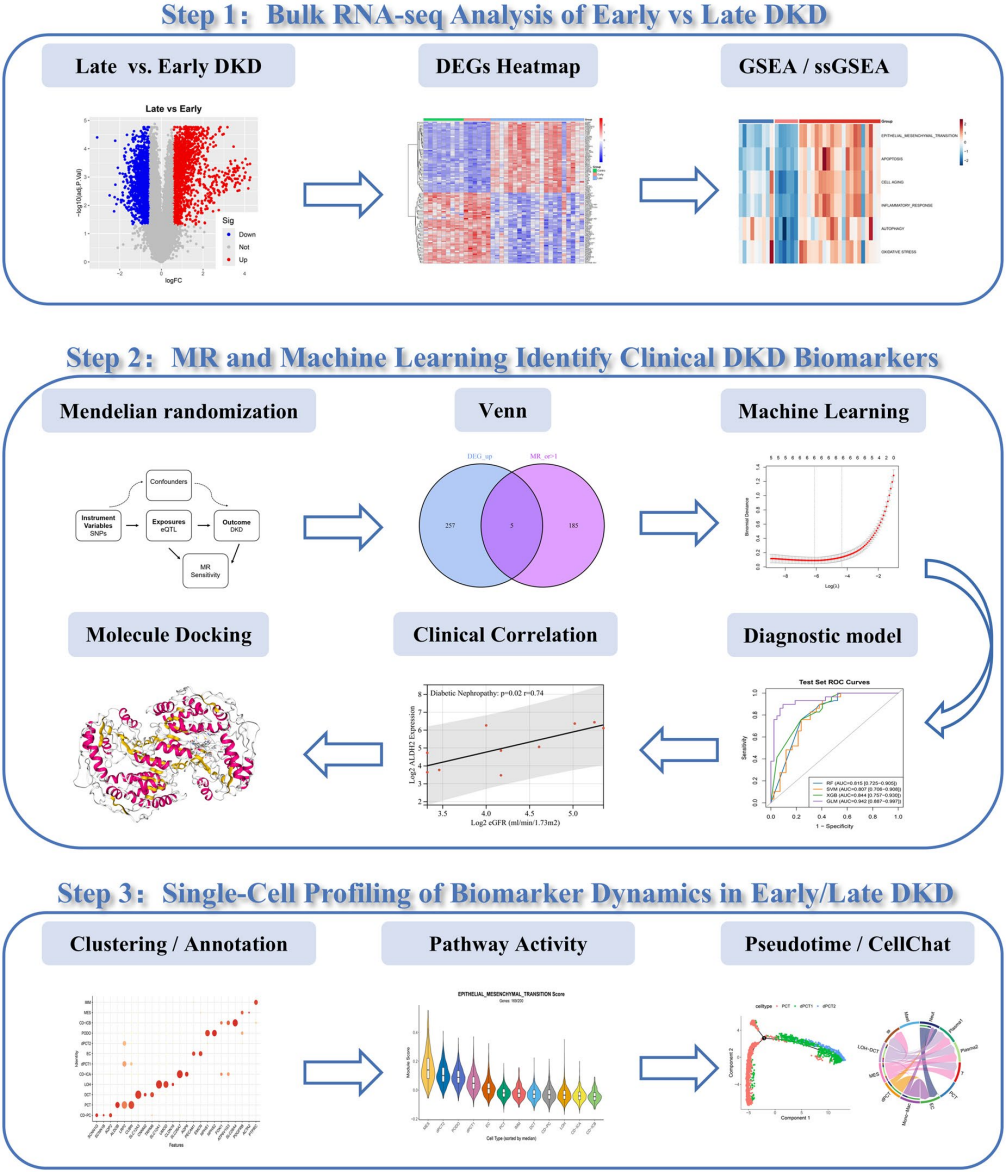
The overall workflow of the study. Abbreviations: DKD, diabetic kidney disease; DEGs, differentially expressed genes; GSEA, Gene set enrichment analysis; ssGSEA, single-sample gene set enrichment analysis; MR, Mendelian randomization.

**Figure 2.**
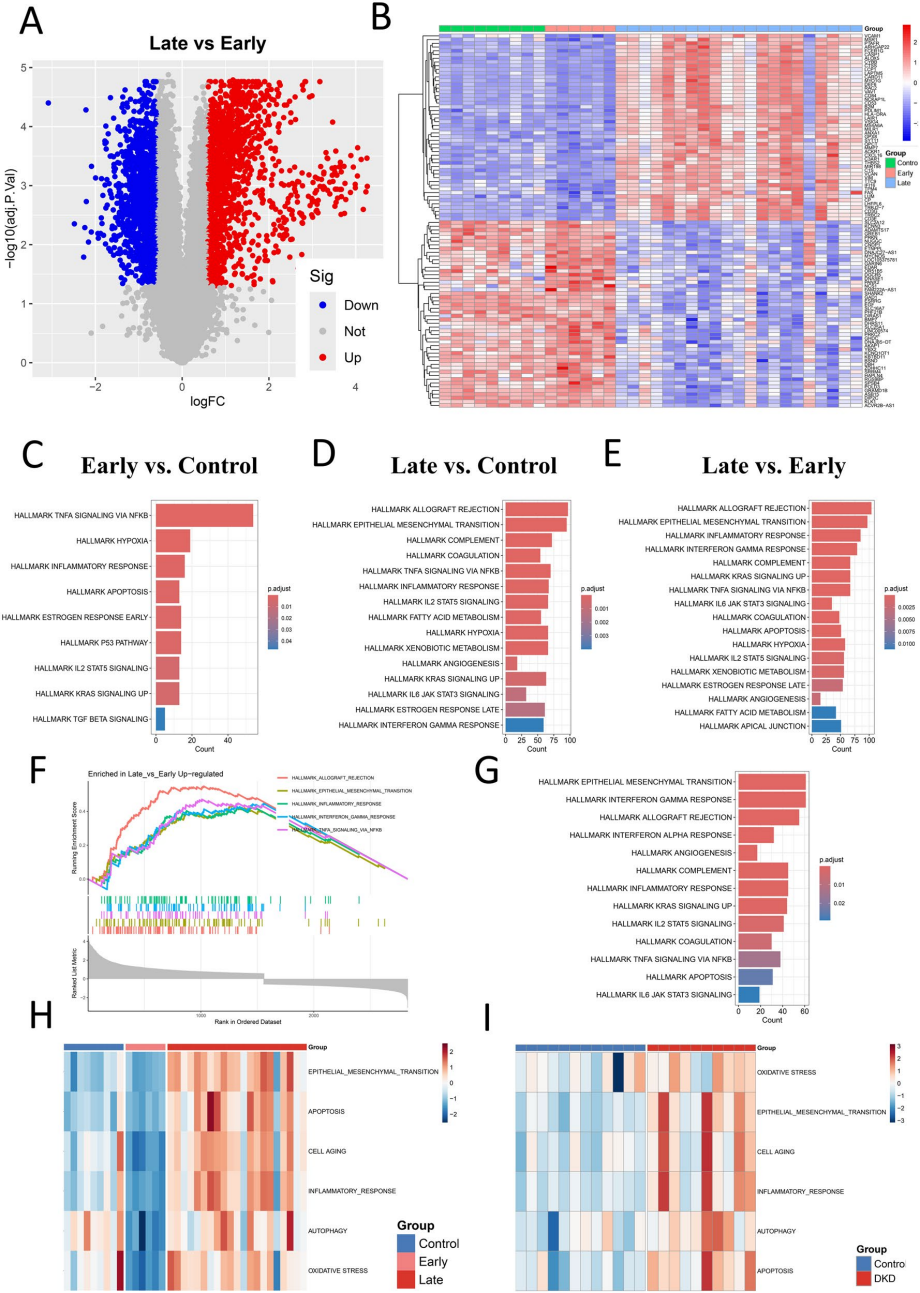
Transcriptomic dysregulation in diabetic kidney disease (DKD) progression. (A) Volcano plot of differentially expressed genes (DEGs) between late and early-stage DKD in GSE142025 (|logFC|>0.585, adjusted P < 0.05). (B) Heatmap of the top 50 DEGs, with samples grouped as healthy controls (green), early-stage DKD (red), and late DKD (blue). (C-E) Hallmark pathway enrichment in GSE142025: (C) early-stage DKD vs. controls, (D) late DKD vs. controls, and (E) late vs. early-stage DKD. (F) GSEA plot of Hallmark pathways in late DKD compared to early-stage DKD. (G) Validation of Hallmark pathways (late DKD vs. controls) in GSE30529. (H-I) ssGSEA scores of pathological pathways across DKD stages in GSE142025 (H) and GSE30529 (I).

An overview of the study design is presented in Figure 1, with the detailed analytical pipeline provided in Supplementary Figure S1. A total of 2,833 DEGs were identified between late and early DKD patients in the GSE142025 cohort, including 1,557 significantly upregulated and 1,276 downregulated genes (|logFC| > 0.585, adjusted *p* < 0.05) (Table S3). These DEGs were visualized *via* volcano plots and heatmaps, with the latter displaying the top 50 most significant genes (Figure 2A,B). Similar analyses revealed 3,525 DEGs between late DKD and controls (Table S4), and 390 DEGs between early DKD and controls (Table S5), demonstrating increasing transcriptional dysregulation with disease progression.

To elucidate the biological mechanisms underlying DKD, we performed pathway enrichment analyses on DEGs. Hallmark analysis showed significant enrichment of apoptosis and inflammatory response in early DKD compared to controls, while EMT, apoptosis, and inflammatory response were more prominent in late DKD, with an increased number of associated genes (Figure 2C–E). GSEA further supported these findings, with high normalized enrichment scores for the same pathways (e.g., EMT; Figure 2F). Independent validation in the GSE30529 dataset confirmed abnormal activation of EMT, inflammatory response, and apoptosis in late DKD, consistent with previous reports [32,33]. Recent studies have increasingly linked oxidative stress, cellular senescence, and autophagy to DKD pathogenesis [34,35]. The ssGSEA revealed significant enrichment of these pathways in late DKD (Figure 2H,I), suggesting their potential contribution to disease progression. These findings highlight the need for single-cell resolution analysis to precisely identify the cell types involved in these pathological processes.

### Mendelian randomization for causal inference in DKD

**Figure 3.**
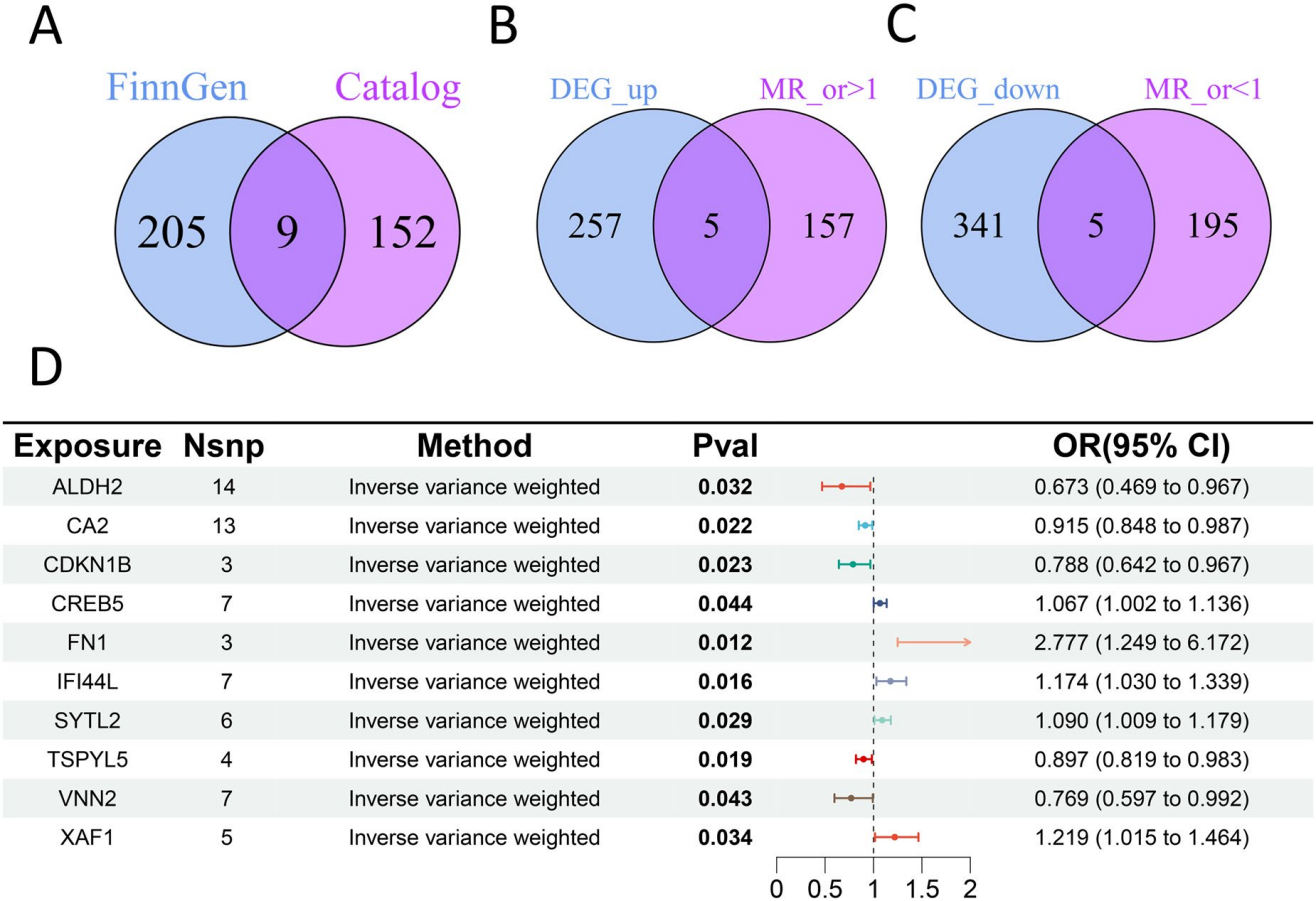
Mendelian randomization (MR) screening for causal genes in DKD. (A) Venn diagram of MR causal genes from FinnGen and GWAS Catalog (366 genes in the union set). (B-C) Identification of 10 key genes by intersecting: (B) MR risk genes with DKD high-expression genes, and (C) MR protective genes with DKD low-expression genes. (D) Forest plot of the 10 key genes showing inverse-variance weighted (IVW) MR results (P < 0.05 indicates statistical significance).

We identified 26,152 independent SNPs associated with 5,430 distinct genes as robust instrumental variables. Using two-sample MR, we assessed the causal effects of these SNPs on DKD based on summary statistics from the FinnGen database and the GWAS Catalog. This analysis revealed 214 DKD-associated genes in FinnGen (Table S6) and 161 in the GWAS Catalog (Table S7). After excluding four genes with inconsistent effect directions between the two datasets, we retained 366 consensus causal genes (Figure 3A).

By intersecting MR-derived causal genes with transcriptomic DEGs, we identified and shortlisted 10 candidate genes: five risk factors (CREB5, FN1, IFI44L, SYTL2, XAF1; IVW OR >1, *p* < 0.05) and five protective factors (ALDH2, CA1, CDKN1B, TSPYL5, VNN2; IVW OR <1, *p* < 0.05) (Figure 3B–D). Detailed SNP information and MR results (IVW, MR-Egger, and weighted median) are provided in Tables S8–S9, respectively. To rigorously validate these findings, we performed comprehensive sensitivity analyses. While funnel plots demonstrated overall bilateral symmetry of SNPs (Figure S2), three genes (CDKN1B, CREB5, and TSPYL5) exhibited relatively weaker symmetry patterns (Figure S2C, D, H). Leave-one-out analyses confirmed no significant influential outliers for most candidate genes (Figure S3), though VNN2 showed marginal sensitivity to individual SNP exclusion (Figure S3I). These genes were further evaluated in downstream validation. Additionally, we observed no evidence of heterogeneity (Table S10), horizontal pleiotropy (Table S11), or reverse causality (Table S12) for the 10 candidate genes.

### A machine learning-based diagnostic model for DKD progression

**Figure 4.**
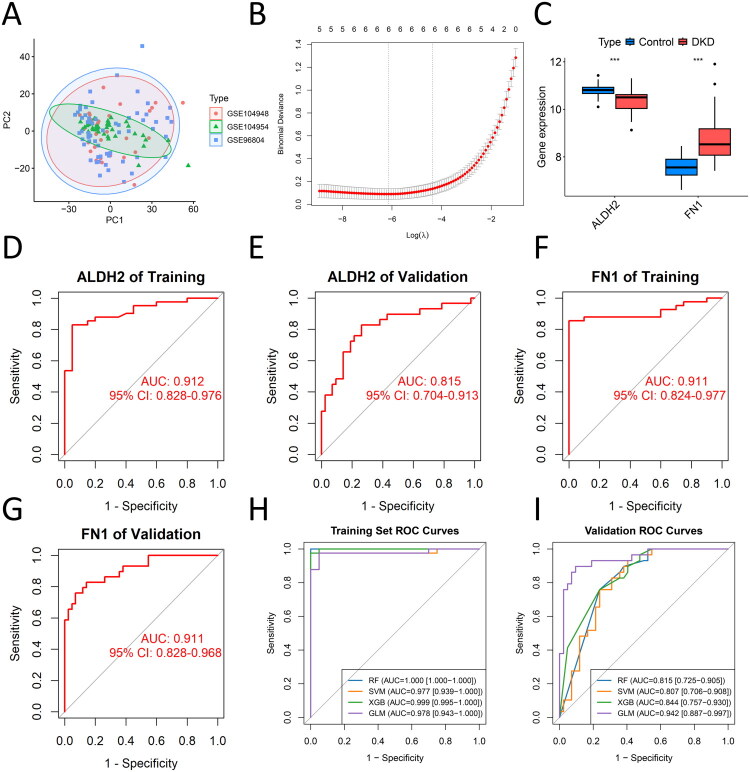
Machine learning-based diagnostic model for DKD. (A) Principal component analysis (PCA) plot after batch-effect correction for training (GSE96804) and validation sets (GSE104948/GSE104954). Each point represents a sample, with the color and shape of the points indicating the source dataset. (B) Least Absolute Shrinkage and Selection Operator (LASSO) regression with 10-fold cross-validation determining the optimal λ. (C) Expression levels of FN1 and ALDH2 in DKD versus controls in the validation dataset (*P < 0.05, **P < 0.01, ***P < 0.001). (D-G) Receiver operating characteristic (ROC) curves of FN1 and ALDH2 in the training set and validation sets. (H-I) Four machine learning approaches evaluating combined FN1 and ALDH2 diagnostic performance in training (H) and validation (I) sets.

Figure 4A displays the principal component analysis (PCA) plot after batch effect correction in the training and validation sets. LASSO regression analysis was performed on the 10 candidate genes, identifying 6 genes (CDKN1B, ALDH2, FN1, XAF1, TSPYL5, and VNN2) (Figure 4B). To evaluate their diagnostic potential, ROC curves were generated for key genes in both the training (GSE96804) and validation (GSE104948/GSE104954) datasets. The ROC analysis revealed that FN1 and ALDH2 exhibited the highest performance, with AUCs > 0.8 in the validation set, respectively, suggesting their roles as potential biomarkers for DKD. Consistent with MR and differential expression analyses, FN1 was upregulated and ALDH2 downregulated in DKD, indicating robust single-gene diagnostic capability (Figure 4C–G). The AUC values of four genes (CDKN1B, TSPYL5, VNN2, XAF1) in the validation set were below 0.7 (Figure S4).

To further improve diagnostic accuracy, we integrated FN1 and ALDH2 using four machine learning algorithms (RF, SVM, GLM, XGBoost). All models achieved AUCs > 0.8 in the validation set (Figure 4H,I). Notably, the GLM model outperformed others, with an AUC of 0.978 (95% CI: 0.943–1.000) in the training set and 0.942 (95% CI: 0.887–0.997) in the validation set, demonstrating that combining both genes enhanced diagnostic power. Genes with less reliable MR evidence (e.g., CDKN1B, CREB5, TSPYL5 with asymmetric funnel plots; VNN2 sensitive to SNP exclusion) were excluded during machine learning validation, strengthening the robustness of our final gene set. The calibration curve demonstrated good overall agreement between predicted probabilities and observed outcomes across most of the risk spectrum (Figure S5A). However, a slight deviation was observed in the mid-risk range (predicted probabilities of 0.4–0.6), where the model marginally underestimated the observed risk. Decision curve analysis confirmed the clinical utility of our model, demonstrating a superior net benefit across most threshold probabilities compared to default strategies (Figure S5B).

### Association between candidate genes and renal function

**Figure 5.**
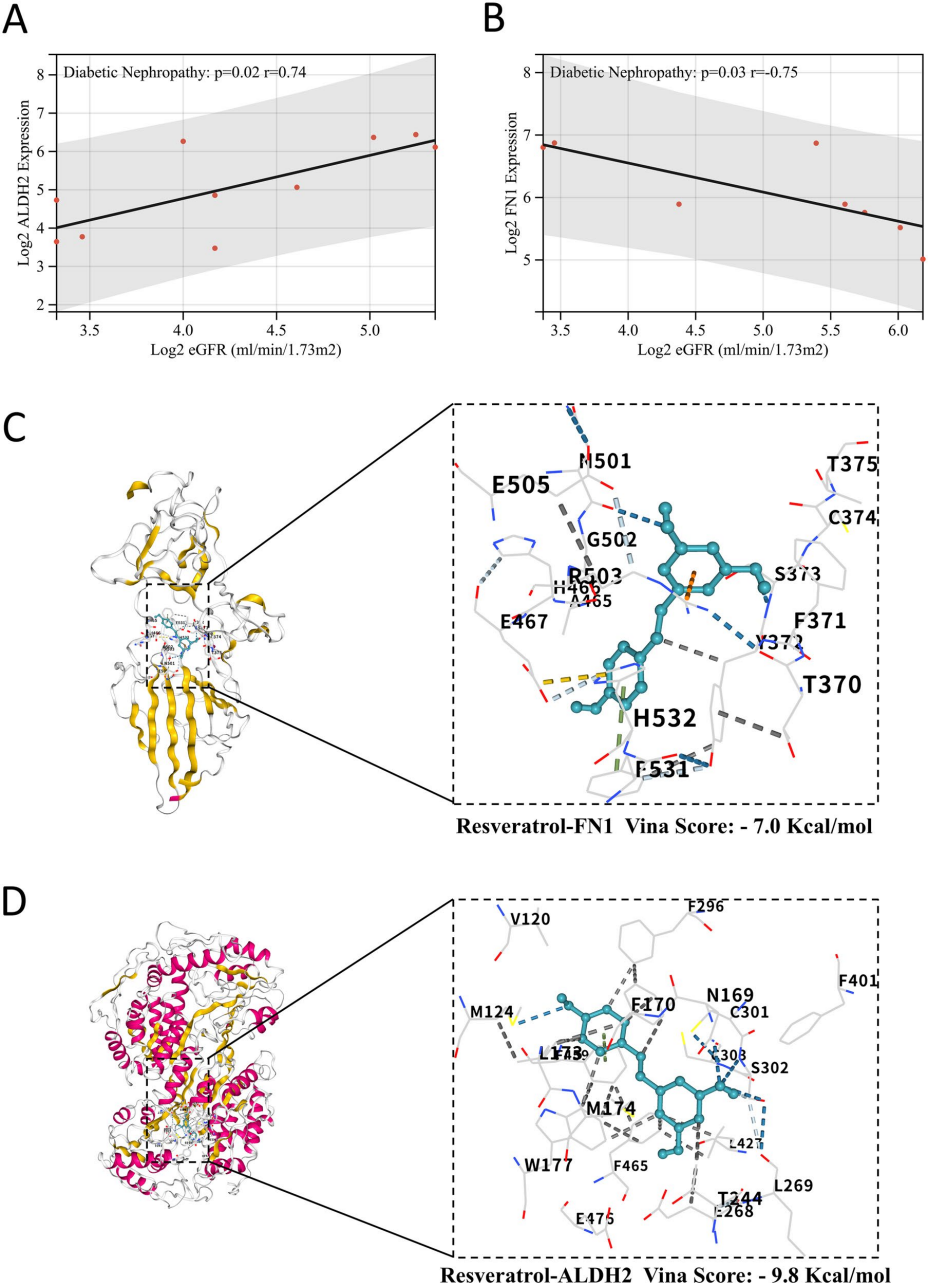
Clinical correlation and molecular docking analysis. (A-B) Pearson correlation analysis of (A) ALDH2 and (B) FN1 with eGFR in the Nephroseq database. (C-D) Molecular docking of resveratrol with (C) FN1 and (D) ALDH2, highlighting predicted binding sites.

Using the Nephroseq database, we analyzed the correlations of ALDH2 and FN1 with eGFR. ALDH2 exhibited a significant positive correlation with eGFR (*r* = 0.74, *p* = 0.02; Figure 5A), while FN1 showed a negative correlation (r = −0.75, *p* = 0.03; Figure 5B). These results support the utility of ALDH2 and FN1 as potential biomarkers for assessing renal function in DKD.

### Docking validation of resveratrol’s FN1-ALDH2 dual targeting

Initial screening identified 79 FN1-reducing and 35 ALDH2-increasing compounds from DSigDB (Table S13). Intersection analysis yielded five dual-target agents: resveratrol, retinoic acid, cyclosporin A, atrazine, and tetradioxin. Based on a comprehensive evaluation of safety profiles and therapeutic potential, resveratrol was selected for further investigation. Molecular docking with the tertiary structures of FN1 (PDB ID: 3m7p) and ALDH2 (PDB ID: 8dr9) confirmed the network pharmacology-predicted interactions, with resveratrol exhibiting strong binding affinities to both targets (Vina score: −7.0 kcal/mol for FN1; −9.8 kcal/mol for ALDH2; Figure 5C,D). These results support resveratrol’s dual-targeting potential for DKD treatment.

### Single-cell transcriptomics of kidney injury

**Figure 6.**
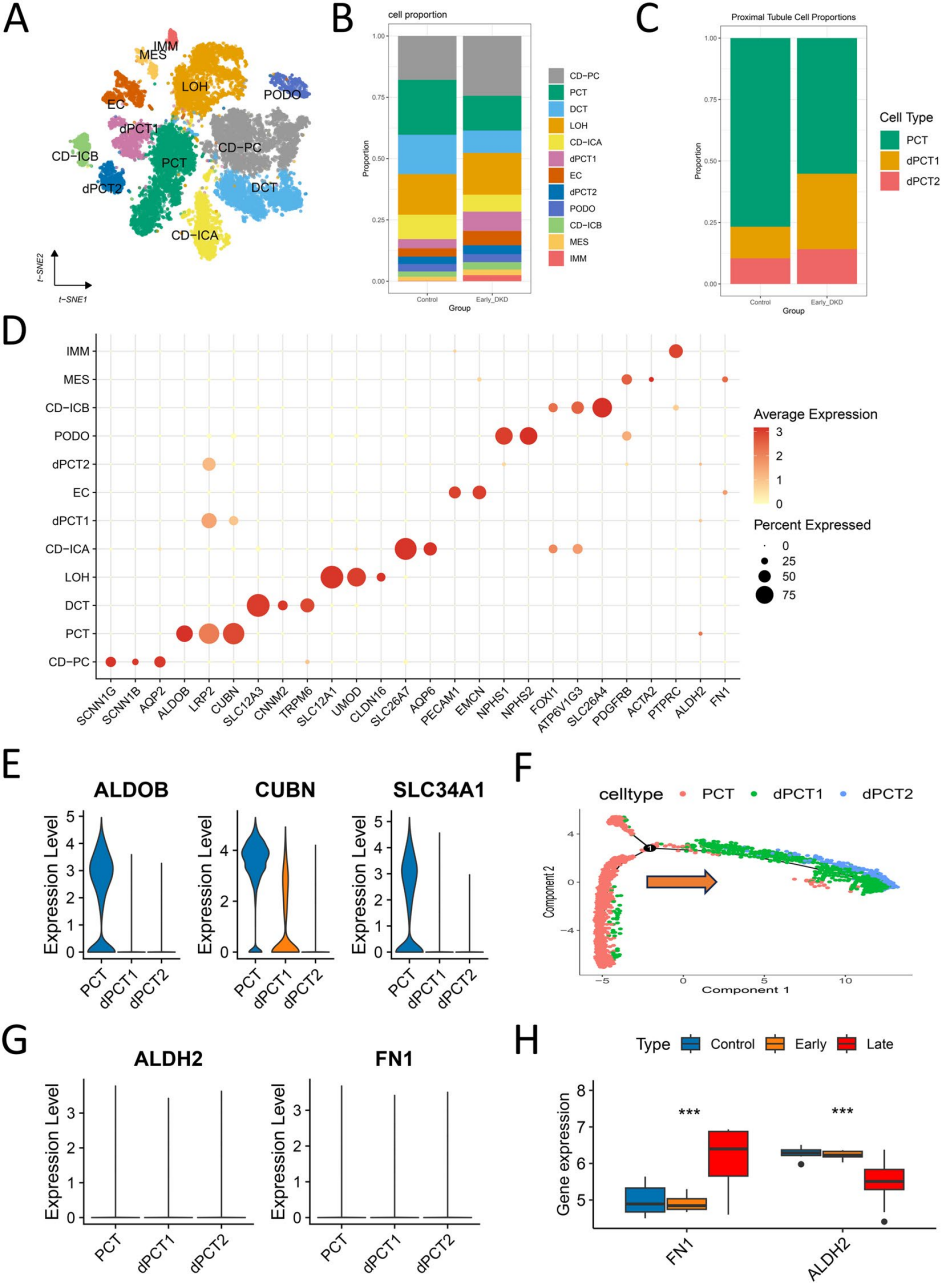
Single-cell atlas of early-stage DKD. (A) t-distributed stochastic neighbor embedding (t-SNE) projection of 12 cell subpopulations in early-stage DKD. (B) Stacked bar plot comparing cell type distributions between early-stage DKD and controls. (C) Compositional distribution of three proximal tubule subtypes (PCT, dPCT1, dPCT2). (D) Bubble plot exhibiting canonical markers for 12 distinct cell types. (E) Violin plots showing the mRNA expression patterns of key functional genes (ALDOB, CUBN, and SLC34A1) across three proximal convoluted tubule (PCT) subtypes: normal PCT, mildly damaged (dPCT1), and severely damaged (dPCT2) cells. The x-axis represents the PCT subtypes with increasing severity of injury, and the y-axis indicates normalized mRNA expression levels. (F) Pseudotime trajectory analysis showing the transition process from normal PCT to damaged dPCT1 and dPCT2 subtypes. The yellow arrow indicates the direction of cellular transformation during the pseudotime process. (G) Violin plots depicting the mRNA expression levels of candidate biomarkers ALDH2 and FN1 across three proximal convoluted tubule (PCT) subtypes. The x-axis represents the PCT subtypes with increasing severity of injury, and the y-axis indicates normalized mRNA expression levels. (H) Differential expression of ALDH2 and FN1 in bulk transcriptomes (early-stage DKD, late-stage DKD vs. controls; *P < 0.05, **P < 0.01, ***P < 0.001).

Following normalized quality control and batch-effect correction using Harmony (v 1.2.3), 20,220 (GSE131882) and 18,816 (GSE209781) high-quality cells were retained for analysis. Using canonical markers [5,36,37] and CellMarker 2.0 [38], we identified 12 cell subsets in early DKD **(**Figure 6A,D**)**, including collecting duct principal cells (CD-PC), proximal convoluted tubule (PCT) cells, distal convoluted tubule (DCT) cells, loop of Henle (LOH), collecting duct intercalated type-A/B (CD-ICA/CD-ICB) cells, endothelial cells (EC), podocytes (PODO), mesangial (MES) cells, immune (IMM) cells, and two damaged PCT subtypes (dPCT1/dPCT2). Comparative analysis revealed significantly expanded dPCT1/dPCT2 populations in early DKD versus controls (Figure 6B, C), despite minimal immune infiltration in both groups. Progressive functional impairment was observed *via* sequential loss of PCT transporters (ALDOB, CUBN, SLC34A1), with dPCT2 showing the most severe depletion (Figure 6E). Pathological pathway activation – encompassing oxidative stress, inflammation, EMT, senescence, and apoptosis – showed hierarchical elevation (dPCT2 > dPCT1 > PCT) *via* AddModuleScore quantification (Figure S6). Pseudotime analysis positioned dPCT1 as intermediate between normal PCT and terminally damaged dPCT2 (Figure 6F), delineating a tubular injury trajectory. Notably, during the early stage of DKD renal tubular damage, ALDH2 and FN1 expression showed no significant changes in early DKD (Figure 6G), aligning with bulk transcriptomics data (GSE142025; Figure 6H).

**Figure 7.**
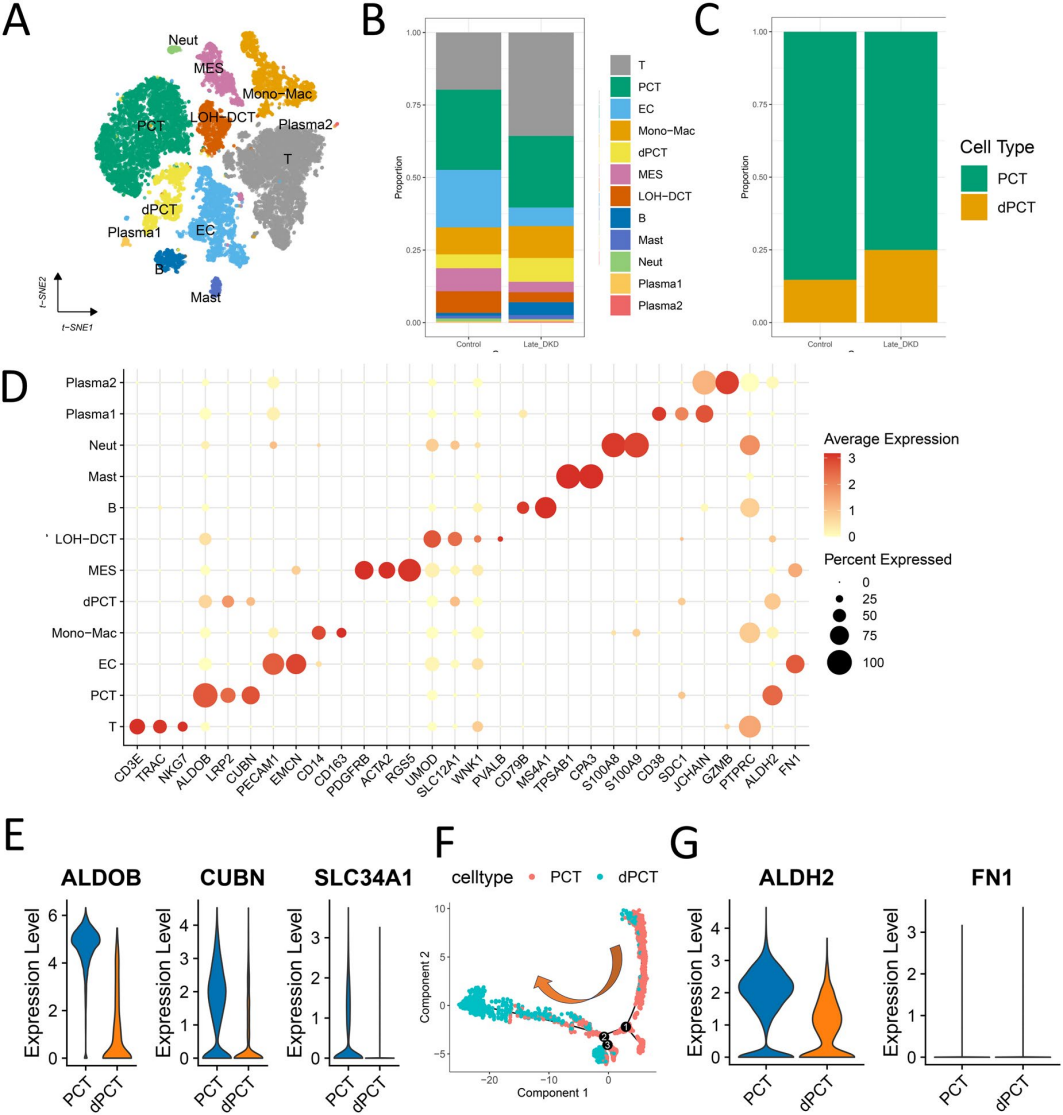
Single-cell atlas of late-stage DKD. (A) t-SNE projection of 12 cellular subpopulations in late-stage DKD. (B) Comparative stacked bar plot of cell type distributions between late-stage DKD and healthy controls. (C) Proportional distribution of two proximal tubule subtypes (PCT and dPCT). (D) Bubble plot displaying canonical markers for 12 distinct cell types. (E) Violin plots showing the mRNA expression patterns of key functional genes (ALDOB, CUBN, and SLC34A1) across three proximal convoluted tubule (PCT) subtypes: normal PCT, and damaged (dPCT) cells. The x-axis represents the PCT subtypes with increasing severity of injury, and the y-axis indicates normalized mRNA expression levels. (F) Pseudotime trajectory analysis demonstrating the transition from normal PCT to damaged dPCT phenotype. The yellow arrow indicates the direction of cellular transformation during the pseudotime process. (G) Violin plots depicting the mRNA expression levels of candidate biomarkers ALDH2 and FN1 across two proximal convoluted tubule (PCT) subtypes. The x-axis represents the PCT subtypes with increasing severity of injury, and the y-axis indicates normalized mRNA expression levels.

In late-stage DKD, clustering resolved 5 renal cell types (endothelial cells, PCT, dPCT, mesangial cells, and LOH-DCT) and 7 immune subtypes (T cells, monocytes/macrophages, B cells, mast cells, neutrophils, plasma1/2) (Figure 7A, D). This stage featured pronounced immune infiltration (Figure 7B) and dPCT expansion (Figure 7C). Key functional markers (ALDOB, CUBN, SLC34A1) were significantly downregulated in dPCT compared to PCT (Figure 7E,F). Pathological pathways (oxidative stress, inflammation, EMT, senescence, apoptosis) showed progressive activation along the PCT-dPCT continuum, with maximal dysregulation in dPCT (Figure S7). In contrast to early DKD, ALDH2 and FN1 expression significantly decreased in dPCT (Figure 7G); these alterations were closely associated with DKD progression to the late stage.

**Figure 8.**
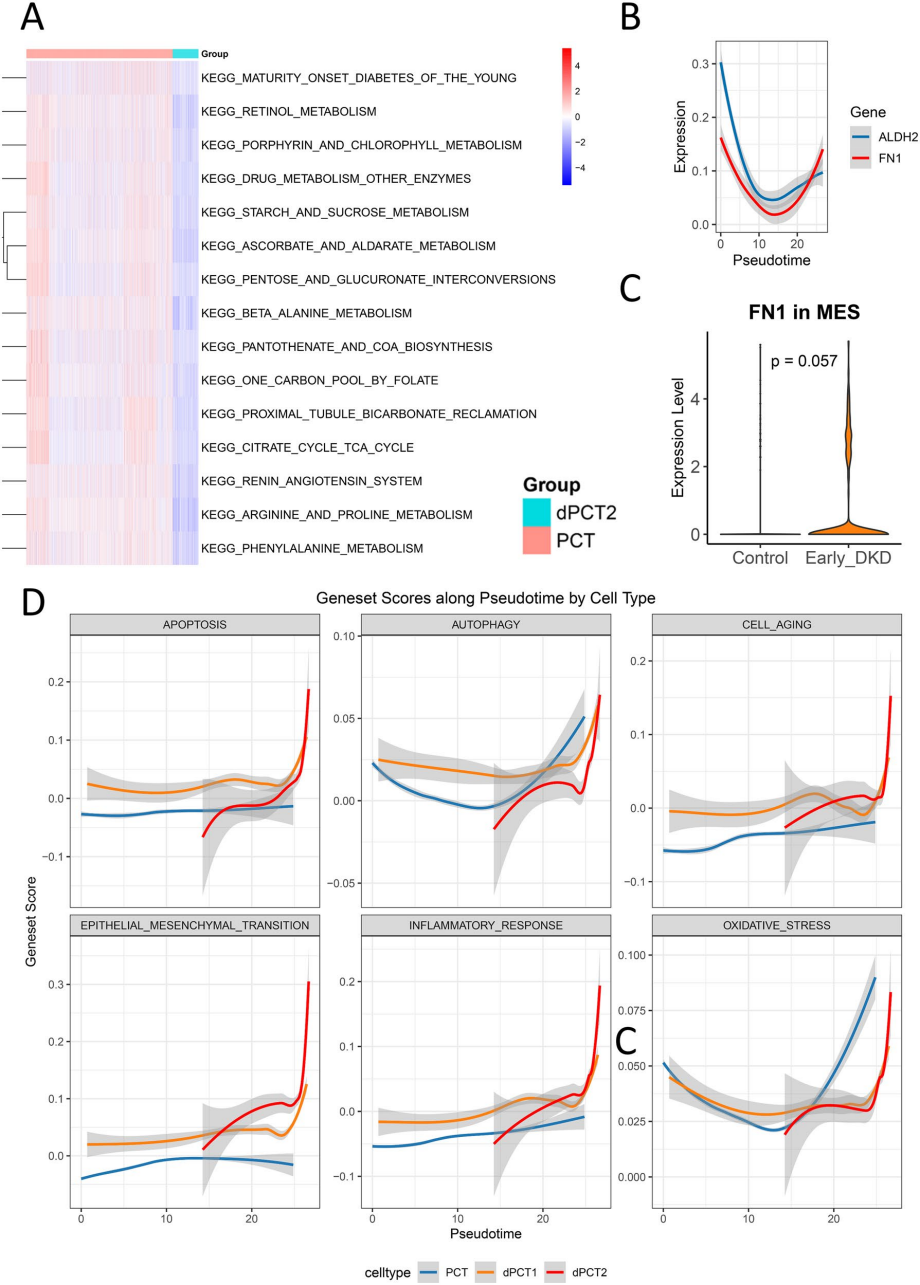
Pseudotime trajectory analysis and pathway dysregulation in early-stage DKD. (A) ssGSEA pathway enrichment heatmap comparing damaged dPCT2 with normal PCT (red indicates significant enrichment in dPCT2; blue indicates enrichment in PCT). (B) Expression trends of ALDH2 and FN1 during PCT-to-dPCT2 pseudotime transition. (C) Comparative analysis of FN1 expression levels in mesangial cells between early-stage DKD patients and healthy controls (violin plots). (D) Trends in enrichment scores of key pathological pathways (apoptosis, autophagy, cellular senescence, epithelial-mesenchymal transition, inflammatory response, oxidative stress) during PCT-to-dPCT2 pseudotime transition.

**Figure 9.**
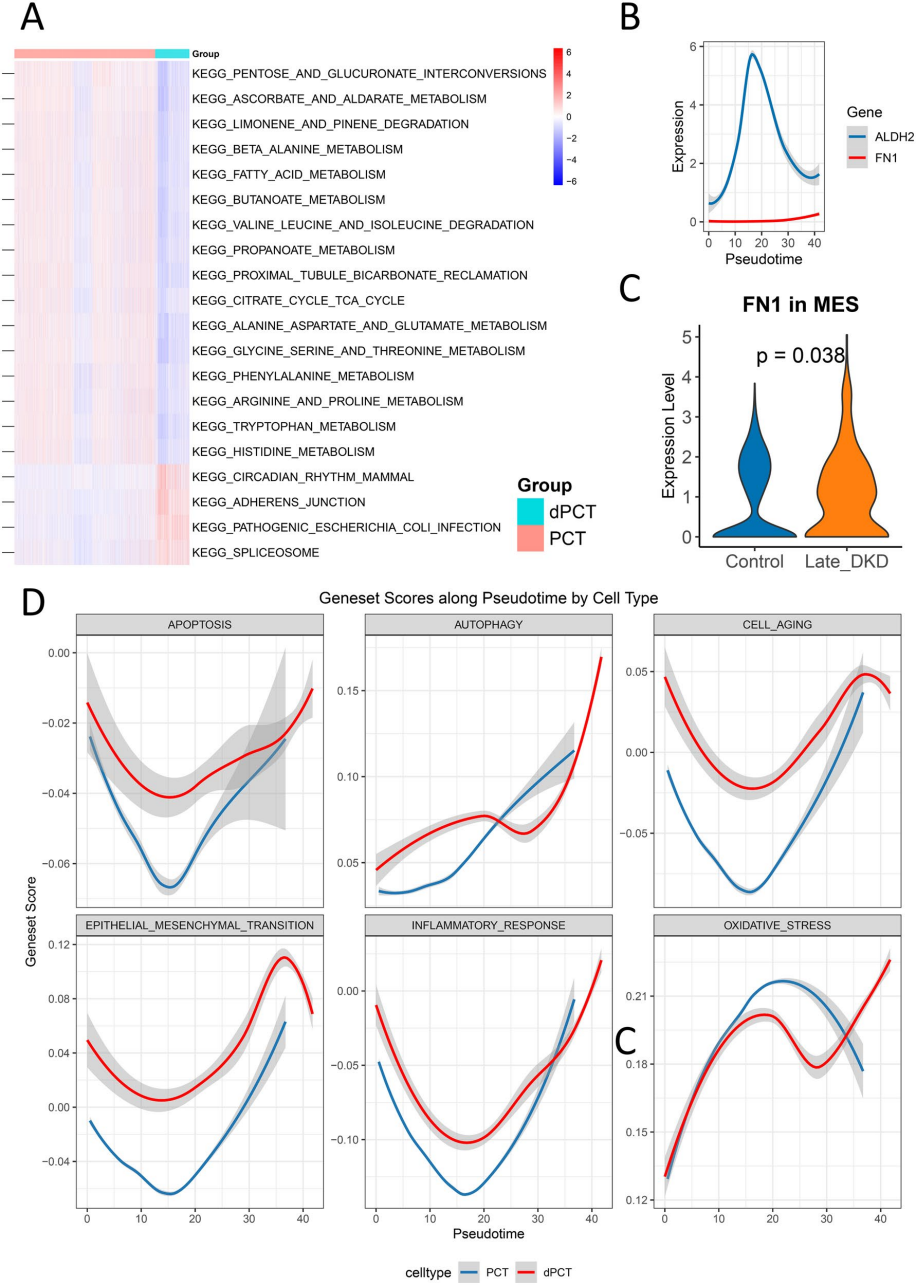
Pseudotime trajectory and pathway dysregulation in late-stage DKD. (A) ssGSEA pathway enrichment heatmap comparing damaged dPCT with normal PCT (red indicates significant enrichment in dPCT; blue indicates enrichment in PCT). (B) Expression trends of ALDH2 and FN1 during PCT-to-dPCT pseudotime transition. (C) Comparative analysis of FN1 expression levels in mesangial cells between late-stage DKD patients and healthy controls (violin plots). (D) Trends in enrichment scores of key pathological pathways (apoptosis, autophagy, cellular senescence, epithelial-mesenchymal transition, inflammatory response, oxidative stress) during pseudotime transition.

To further compare the molecular features between intact PCT and dPCT across early and late disease stages. ssGSEA of 186 KEGG gene sets uncovered stage-dependent molecular alterations in dPCT. Early (Figure 8A) and late-stage dPCT (Figure 9A) consistently showed impaired energy metabolism, disrupted amino acid metabolism (including glycine and arginine pathways), and compromised bicarbonate reabsorption function. In late-stage dPCT, we observed unique pathway enrichments, particularly in pathogenic Escherichia coli infection and adherens junction signaling. These findings may suggest that advanced dPCT develops chronic inflammatory characteristics and potentially experiences enhanced EMT processes.

### Temporal expression patterns of ALDH2 and FN1 across DKD stages

In early-stage DKD, pseudotime analysis of ALDH2 and FN1 in PCT and dPCT1/2 cells during early DKD showed that FN1 upregulation began precisely when ALDH2 expression declined to its lowest point (Figure 8B). The elevated FN1 expression coincided with activated pathological pathways (including cellular senescence, inflammatory response, EMT, and apoptosis) in dPCT2 (Figure 8D), suggesting a potential association between FN1 expression and these injury-related processes. Notably, although MES displayed the highest EMT scores (Figure S6C), FN1 expression in MES showed no statistically significant elevation compared to controls (*p* > 0.05) (Figure 8C).

Regarding late-stage DKD, pseudotime analysis of ALDH2 and FN1 in PCT/dPCT revealed distinct expression dynamics: ALDH2 exhibited an initial increase followed by progressive decline during PCT-to-dPCT transition, while FN1 remained stably low (Figure 9B). Notably, ALDH2 expression demonstrated an inverse relationship with pathological pathway activation in dPCT (Figure 9D), indicating ALDH2 may negatively regulate cellular senescence, inflammatory response, EMT, and apoptosis in late-stage DKD. These observations imply a transient protective response through ALDH2 upregulation that ultimately fails to counteract disease progression, resulting in sustained pathological pathway activation. Furthermore, MES showed both the highest EMT enrichment scores (Figure S7C) and significantly elevated FN1 expression versus controls (*p* < 0.05, Figure 9C). This marked contrast with early DKD findings indicates FN1 may contribute to advanced-stage-specific fibrotic transformation in MES, providing new clues to late DKD pathogenesis. Analysis of urinary single-cell RNA-seq data revealed significantly elevated expression of FN1 and ALDH2 in late-stage versus early-stage DKD (*p* < 0.05) (Figure S8).

### Divergent cell-cell interaction patterns in early versus late DKD

Intercellular communication patterns were systematically compared between early and late DKD stages using the CellchatDB algorithm. Quantitative analysis revealed significantly enhanced interaction numbers and intensity in advanced DKD compared to normal controls (Figures S9A and S10A). Heatmap characterization uncovered stage-specific features: while PCT maintained active crosstalk with other cells in early DKD, their damaged counterparts (dPCT1/2) showed markedly reduced interactions (Figure S9B), likely reflecting structural or functional impairment. In contrast, late-stage DKD exhibited prominent immune cell-centric communication networks due to extensive immune infiltration, particularly between tubular and immune compartments (Figure S10B).

Ligand-receptor analysis revealed distinct interaction modules (Figures S9C and S10C). Late-stage dPCT predominantly interacted with immune cells (monocytes/macrophages, plasma cells, B cells, and T cells) *via* MIF- and SPP1-related pathways, potentially contributing to disease pathogenesis.

## Discussion

### Integrated multi-omics validation of FN1 and ALDH2 in DKD

The global burden of chronic kidney disease (CKD) continues to escalate, currently representing the seventh leading mortality risk factor worldwide and projected to become the fifth leading cause of years of life lost by 2040[39]. As the predominant etiology of CKD and end-stage kidney disease (ESKD) [1], DKD presents complex molecular pathogenesis and critically lacks reliable biomarkers for early detection. This clinical challenge has gained renewed urgency since the World Health Organization’s (WHO) recent landmark decision to prioritize kidney diseases among non-communicable diseases (NCDs) contributing to premature mortality, emphasizing the imperative for enhanced early screening and targeted therapies [40]. In this study, we implemented a robust integrated validation pipeline combining bulk and single-cell transcriptomics, Mendelian randomization, machine learning, and molecular docking. This approach allowed us to rigorously verify and establish the roles of previously implicated molecules, specifically FN1 and ALDH2, as robust, stage-specific biomarkers in DKD progression, rather than discovering them anew. Our systematic multi-omics profiling confirmed FN1 as a risk factor and ALDH2 as a protective factor. The novelty of our work lies in the comprehensive and cross-validated nature of this analytical framework, which provides a higher level of evidence for the diagnostic utility of these biomarkers across different disease stages. Importantly, our framework may be extended to other chronic kidney diseases and precision medicine initiatives.

### Single-cell trajectories of progressive tubular injury

The pathogenesis of DKD involves complex interactions between multiple pathological processes. Studies have demonstrated tubular epithelial cell dysfunction, accumulation of cellular senescence markers (e.g., increased SA-β-galactosidase activity and upregulated Cdkn2a, Cdkn1a expression) [41], and aggravated cellular damage manifestations, including DNA damage and oxidative stress during DKD progression [42]. These changes are accompanied by increased apoptosis and pro-inflammatory factor release, further leading to plasma membrane damage and cytoskeleton disruption in tubular epithelial cells [43]. With disease progression, sustained inflammatory responses and excessive extracellular matrix deposition jointly contribute to the formation of renal fibrotic microenvironment [34]. Notably, the EMT process is also involved, characterized by loss of epithelial polarity and acquisition of mesenchymal phenotype, which may accelerate destruction of renal parenchyma structure [6]. These pathological mechanisms interact with and reinforce each other, ultimately leading to progressive renal function deterioration.

Previous studies classified proximal tubules into seven discrete subtypes based on segment-specific markers (e.g., Slc34a1+) and injury biomarkers, including healthy S1/S2 segments, injured S1/S3 segments (Havcr1+), maladaptive cells (Fxyd5+), newly injured cells (Krt20+), and proliferative populations (Mki67+). This classification system revealed that maladaptive proximal tubular cells exhibit the most pronounced pro-inflammatory and pro-fibrotic characteristics, whereas newly injured proximal tubular cells (Krt20+) paradoxically demonstrate lower fibrotic activity [44].

In contrast, our DKD analysis captured a progressive continuum of PCT dysfunction characterized by sequential loss of key transporters (ALDOB, CUBN, SLC34A1) and hierarchical activation of pathological pathways (oxidative stress, inflammation, EMT, senescence). Pseudotime trajectory analysis further delineated transitional states from normal PCT to mildly damaged (dPCT1) and terminally impaired (dPCT2) cells, providing dynamic insights into chronic disease progression. Notably, the maladaptive PTCs in AKI—marked by prominent inflammatory and fibrotic features [45]—functionally correspond to our early-stage dPCT2 or late-stage dPCT populations in DKD, suggesting shared terminal phenotypes between acute and chronic kidney injury. Moreover, the intermediate dPCT1 population in early-stage DKD may delineate a unique therapeutic window specific to DKD progression. Conversely, the newly injured Krt20+ proximal tubular cells in AKI showed even lower pro-fibrotic scores than healthy cells, reflecting AKI-specific injury response patterns. Additionally, our focus on apoptosis and senescence pathways complements the conventional AKI research perspective centered on inflammation and fibrosis.

### FN1: from renal biomarker to pan-fibrotic mediator

Fibrosis, a hallmark of numerous chronic pathological conditions, stems from dysregulated extracellular matrix (ECM) deposition and remodeling. Fibronectin (FN1), a key ECM component, has been widely associated with fibrosis in various organs, including renal, pulmonary, cardiac and hepatic fibrosis [46–50]. Notably, Lan et al. identified a distinct subset of pro-fibrotic macrophages (Fn1+/Spp1+) in fibrotic cardiac grafts, which promote collagen deposition and ECM remodeling by activating fibroblasts, thereby sustaining a pro-fibrotic microenvironment [51]. These findings highlight FN1 as a critical mediator of fibrotic progression. In DKD, FN1 dysregulation is particularly pronounced. A previous study reported significantly higher urinary Fibronectin levels in diabetic patients, especially those without overt albuminuria [52]. Our study strengthens its candidacy as a biomarker by precisely defining its stage-specific dysregulation. Specifically, single-cell RNA sequencing of DKD patient urine samples revealed elevated FN1 expression in advanced DKD compared to early-stage cases (*p* < 0.05), correlating with disease severity. At the renal single-cell level, advanced DKD exhibited marked FN1 upregulation in MES (*p* < 0.05), alongside the highest EMT score. This suggests that MES may emerge as a dominant effector cell type in FN1-mediated fibrosis during late-stage DKD. Collectively, these findings position FN1 not only as a promising biomarker but, more importantly, as an exemplar of the pro-fibrotic endgame in DKD and a pan-fibrosis marker, given its involvement in dysregulated ECM remodeling common to many organs.

### ALDH2: a causal antioxidant and therapeutic target

ALDH2, a mitochondrial matrix-localized tetrameric enzyme and core member of the ALDH family, plays a crucial role in detoxification by catalyzing the oxidation of reactive aldehydes to less toxic carboxylic acids. This enzymatic activity prevents the formation of deleterious adducts between aldehydes and cellular macromolecules, thereby protecting against genotoxic and cytotoxic damage [53,54]. As a central component of the cellular defense network against oxidative stress and carbonyl stress, ALDH2’s protective role extends beyond DKD to various pathological conditions, including multiple types of CKD, acute kidney injury [55–57], liver fibrosis [58], and cardiovascular diseases [59]. Detailed mapping of renal ALDH2 distribution reveals predominant expression in the proximal tubule, with markedly lower levels in the LOH and CD [60]. This compartmentalized expression pattern aligns with proximal tubule cells’ high mitochondrial density and intense metabolic activity. Functionally, ALDH2 serves as a critical guardian in these energetically demanding cells by maintaining aldehyde homeostasis, whereas its deficiency renders proximal tubule cells particularly susceptible to hypoxic, ischemic, and metabolic insults [61]. The pathological relevance of this mechanism is underscored by aristolochic acid-induced fibrosis models, where ALDH2 downregulation correlates with elevated FN1 expression. Our findings, particularly the MR analysis establishing a causal protective role, solidify ALDH2’s status as an exemplar for early DKD pathophysiology, demonstrating its inverse correlation with renal function.

Single-cell resolution analysis demonstrates significantly reduced ALDH2 expression in damaged PCT cells from advanced DKD patients compared to their normal counterparts. Conversely, urinary sediment single-cell RNA sequencing reveals elevated ALDH2 levels in late-stage DKD patients versus early-stage cases. This apparent discrepancy may reflect either injury-induced ALDH2 leakage from compromised tubules or compensatory secretory mechanisms in advanced disease. The progression of renal injury in advanced Diabetic Kidney Disease (DKD) is characterized by extensive tubular epithelial cell necrosis and detachment, leading to a marked increase in urinary exfoliated cells and cellular debris. This pathological state results in substantial leakage of intracellular components, including ALDH2 mRNA, into the tubular lumen [62,63]. Beyond passive release, we hypothesize a compensatory secretory mechanism may contribute to elevated urinary ALDH2. Analogous to the documented tubular secretion of Klotho [64], renal epithelial cells may actively export ALDH2 under pathological stress to mitigate luminal aldehyde toxicity and maintain microenvironmental homeostasis. This speculative yet physiologically plausible mechanism warrants experimental validation. Furthermore, this distinct behavior positions urinary ALDH2, along with FN1, as a promising noninvasive liquid biopsy marker [65], while their tissue-specific expression patterns offer potential as digital pathology signatures for staging DKD.

### Therapeutic implications and repurposing of resveratrol

Molecular docking demonstrated that resveratrol could target FN1 in DKD treatment. This computational evidence aligns remarkably well with existing experimental findings. Qiao et al. established that resveratrol significantly suppresses high glucose-induced mesangial cell proliferation in diabetic nephropathy models by downregulating the p38 MAPK/TGF-β1 signaling pathway, consequently reducing FN1 secretion and ameliorating glomerulosclerosis and fibrosis [66]. Complementing these findings, Huang et al. demonstrated that resveratrol activates the Sirt1/Nrf2 antioxidant pathway to scavenge reactive oxygen species and prevent advanced glycation end product-induced FN1 overexpression in mesangial cells – the primary effector cells for pathological FN1 secretion, as confirmed by their study and corroborated by our observation of FN1 overexpression in these cells [67].

At the molecular level, spectroscopic analyses by Su et al. revealed that resveratrol induces electron delocalization of aldehyde groups, thereby reducing their chemical reactivity. This unique property enables effective protection of mitochondrial DNA from aldehyde attacks, preventing the formation of mutagenic adducts [68]. Building upon this discovery, researchers developed an optimized acetyl-resveratrol derivative that not only retains the prototype molecule’s aldehyde-detoxifying capacity but also significantly enhances the catalytic efficiency of mitochondrial ALDH2 through modulation of aldehyde electronic states, facilitating the conversion of toxic aldehydes into less harmful carboxylic acid derivatives [69]. Importantly, our molecular docking results corroborate these findings. These significant discoveries provide a solid theoretical foundation for the therapeutic application of resveratrol and its derivatives in DKD treatment. Beyond providing a mechanistic rationale, our study underscores the considerable translational promise of resveratrol. Its favorable safety profile and established use as a nutraceutical lower the barrier for clinical investigation, positioning it as an attractive candidate for pharmacological repositioning in DKD [70]. This repurposing strategy could significantly truncate the developmental timeline by leveraging existing safety data. Moreover, the dual-targeting action of resveratrol—concurrently mitigating FN1-driven fibrosis and augmenting ALDH2-mediated cytoprotection—aligns with the tenets of systems pharmacology, offering a network-based therapeutic strategy for a multifactorial disease. Future research should prioritize *in vivo* validation of resveratrol’s efficacy in progressive DKD models and explore its potential synergism with current standard-of-care agents, such as SGLT2 inhibitors.

### Broader research context and limitations

Our findings should be interpreted within the broader context of rapidly evolving biomedical research trends. In precision medicine, our identification of stage-specific biomarkers supports the shift from universal staging metrics (e.g., eGFR, albuminuria) toward molecularly stratified interventional strategies [71]. As the development of noninvasive diagnostic markers for chronic kidney disease progression advances [52,72,73], our work contributes to this endeavor by nominating potential candidate molecules. Furthermore, our study underscores the pivotal value of single-cell nephrology; by resolving molecular expression features at single-cell resolution, it allows the exploration of whether key diagnostic biomarkers are altered within specific pathological subpopulations [62,74]. Additionally, single-cell spatial transcriptomics, as a powerful tool, holds future promise for elucidating the spatial localization and cell-cell communication of these markers within the tissue microenvironment [75], thereby deepening the understanding of DKD pathogenesis. Meanwhile, artificial intelligence (AI) and machine learning are surpassing traditional statistics by integrating high-dimensional data to detect complex patterns [76–78], empowering tools like radiomics and pathomics for disease identification [79]. Our work demonstrates how AI-driven analysis distills efficient biomarker combinations from multi-omics data, supporting clinically applicable predictive tool development.

This study demonstrates how combining machine learning, Mendelian randomization, and single-cell RNA sequencing enables precision nephrology for diabetic kidney disease. However, the present study has limitations that highlight several areas for further research. Although we integrated renal transcriptome sequencing, single-cell sequencing, and urinary sediment single-cell sequencing data from patients with both early- and late-stage DKD—with standardized quality control and batch correction—the reliance on public databases may still introduce sample heterogeneity, potentially influencing the results. To address this, future studies should uniformly collect and analyze prospective biological samples (e.g., blood, urine, and renal tissue) from the same patient cohort, thereby reducing heterogeneity. Furthermore, while multi-omics analyses revealed the key roles of ALDH2 and FN1 in DKD, their specific mechanisms in disease progression require elucidation through cellular and animal experiments. Such efforts could provide a foundation for developing more effective therapies. Finally, our MR analysis was primarily based on GWAS data from European-ancestry populations. While this minimizes population stratification bias, it limits the generalizability of our findings to other ethnic groups. Future studies are needed to validate the causal roles of FN1 and ALDH2 in diverse populations. In summary, although this study supports the therapeutic potential of ALDH2 and FN1, their clinical applicability awaits validation through translational research.

## Conclusion

In conclusion, our integrated multi-omics approach establishes FN1 and ALDH2 not merely as diagnostic biomarkers, but as stage-specific exemplar genes that epitomize the fibrotic and oxidative stress axes of DKD progression, respectively. This exemplar-based framework provides a molecular taxonomy for DKD that enhances staging precision and paves the way for targeted therapeutics, such as the dual-targeting agent resveratrol identified here.

## Supplementary Material

Tables S1_S13.xlsx

Figures S1_S10.docx

## Data Availability

The analysis code generated in this study has been deposited in the GitHub repository: https://github.com/ljw71865/R-scripts-for-pipeline-reproducibility. The original gene lists, Mendelian randomization results, pathway enrichment results, and supplementary figures and tables have been deposited in the Figshare repository under accession DOI: https://doi.org/10.6084/m9.figshare.30190087. The publicly available datasets re-analyzed in this study include transcriptomic data from the GEO repository (see Supplementary Table S1 for details), FinnGen GWAS summary statistics (accessible at https://storage.googleapis.com/finngen-public-data-r12/summary_stats/release/finngen_R12_DM_NEPHROPATHY_EXMORE.gz), and CKDGen consortium eGFR GWAS summary statistics (accessible at http://ftp.ebi.ac.uk/pub/databases/gwas/summary_statistics/GCST90435001-GCST90436000/GCST90435706/). All other data supporting the findings of this study are available within the main article and its supplementary materials. Additional data requests can be directed to the corresponding authors.

## Abbreviations

DKD: diabetic kidney disease
SGLT2: sodium-glucose cotransporter-2
EMT: epithelial-mesenchymal transition
eGFR: estimated glomerular filtration rate
MR: Mendelian randomization
GEO: Gene Expression Omnibus
GWAS: genome-wide association study
eQTL: expression quantitative trait loci
LD: linkage disequilibrium
KEGG: Kyoto Encyclopedia of Genes and Genomes
MSigDB: Molecular Signatures Database
DEGs: Differentially expression genes
FDR: false discovery rate
GSEA: Gene set enrichment analysis
ssGSEA: single sample Gene set enrichment analysis
IVW: inverse variance-weighted
ROC: receiver operating characteristic
XGBoost: eXtreme Gradient Boosting
SVMRF: support vector machines random forest
GLM: generalized linear model
t-SNE: t-distributed Stochastic Neighbor Embedding
PCA: principal component analysis
CD-PC: collecting duct principal cell
PCT: proximal convoluted tubule
DCT: distal convoluted tubule
dPCT: damaged PCT
IMM: immune
MES: mesangial
PODO: podocytes
EC: endothelial cell
CD-ICA: collecting duct intercalated type-A
CD-ICB: collecting duct intercalated type-B
LOH: loop of Henle
CKD: chronic kidney disease
ESKD: end-stage kidney disease
WHO: World Health Organization
NCDs: non-communicable diseases
ECM: extracellular matrix
