# Supplementary material for: Multi-omics and machine learning identify FN1 and ALDH2 as diagnostic biomarkers and therapeutic targets in early and late diabetic kidney disease
Source: Ren Fail. 2025 Oct 29;47(1):2577849. doi: 10.1080/0886022X.2025.2577849 (PMC12573553; doi:10.1080/0886022X.2025.2577849)
Supplement: Figures S1_S10.docx [file IRNF_A_2577849_SM1707.docx]

**Supplementary Figures**

Figure S1. Schematic of the integrated multi-omics analysis pipeline.

Figure S2. Funnel plot sensitivity analysis for Mendelian randomization.

Figure S3. Leave-one-out sensitivity analysis for Mendelian randomization.

Figure S4. Diagnostic performance of secondary candidate genes.

Figure S5. Model validation using calibration and decision curve analysis.

Figure S6. Pathological pathway activation across cell subpopulations in early-stage DKD.

Figure S7. Pathological pathway activation across cell subpopulations in late-stage DKD.

Figure S8. Urinary single-cell exploration of ALDH2 and FN1 in DKD.

Figure S9. Cell-cell communication network using CellChat in early-stage DKD.

Figure S10. Cell-cell communication network using CellChat in late-stage DKD.


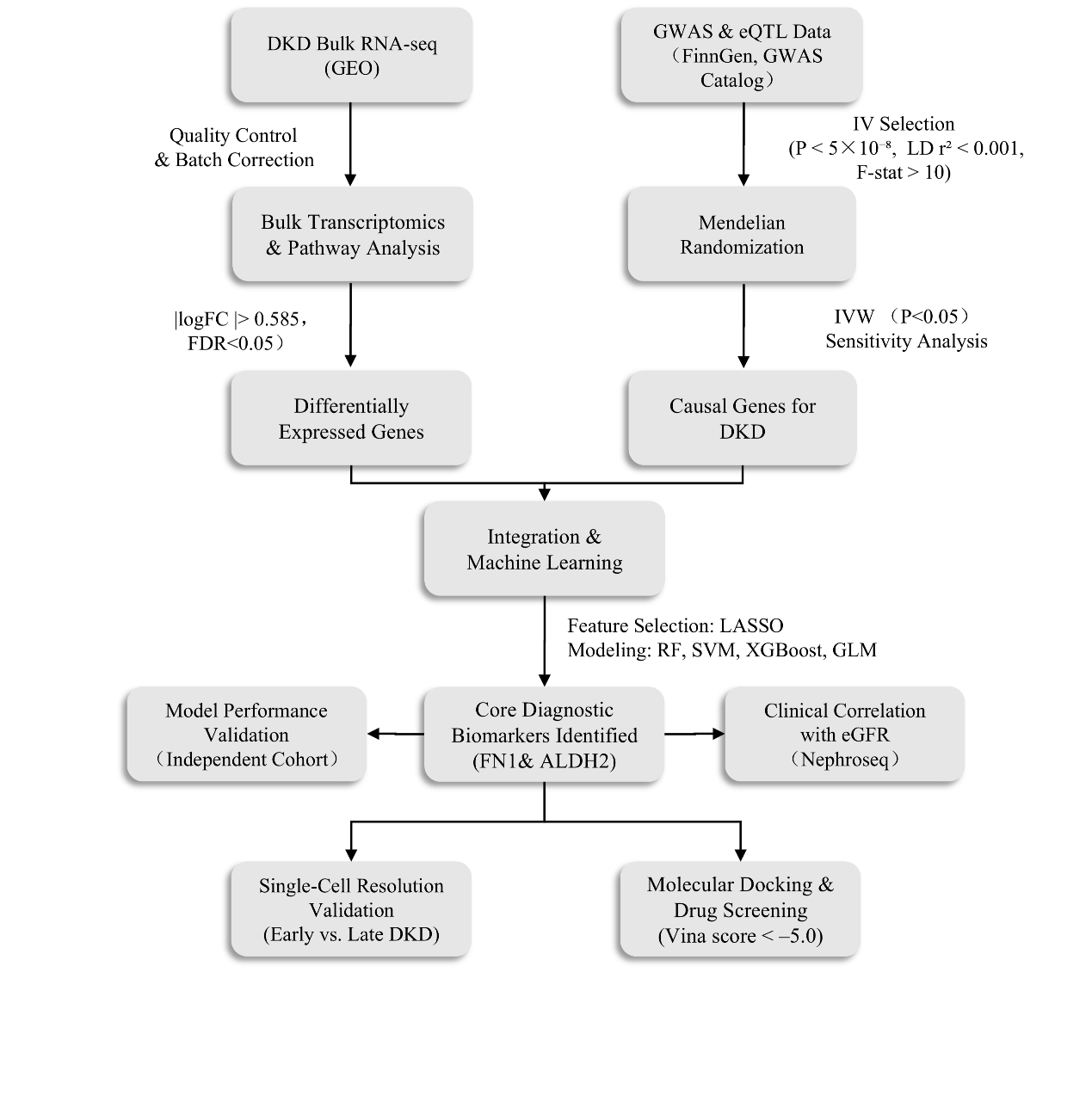


**Figure S1: Schematic of the integrated multi-omics analysis pipeline.** Abbreviations: DKD, diabetic kidney disease; GEO, Gene Expression Omnibus; GWAS, expression quantitative trait loci; eQTL, expression quantitative trait loci; FDR, false discovery rate; IV, instrumental variable; IVW, inverse variance-weighted; LASSO, Least Absolute Shrinkage and Selection Operator; RF, random forest; SVM, support vector machines; XGBoost, eXtreme Gradient Boosting; GLM, Generalized Linear Model.


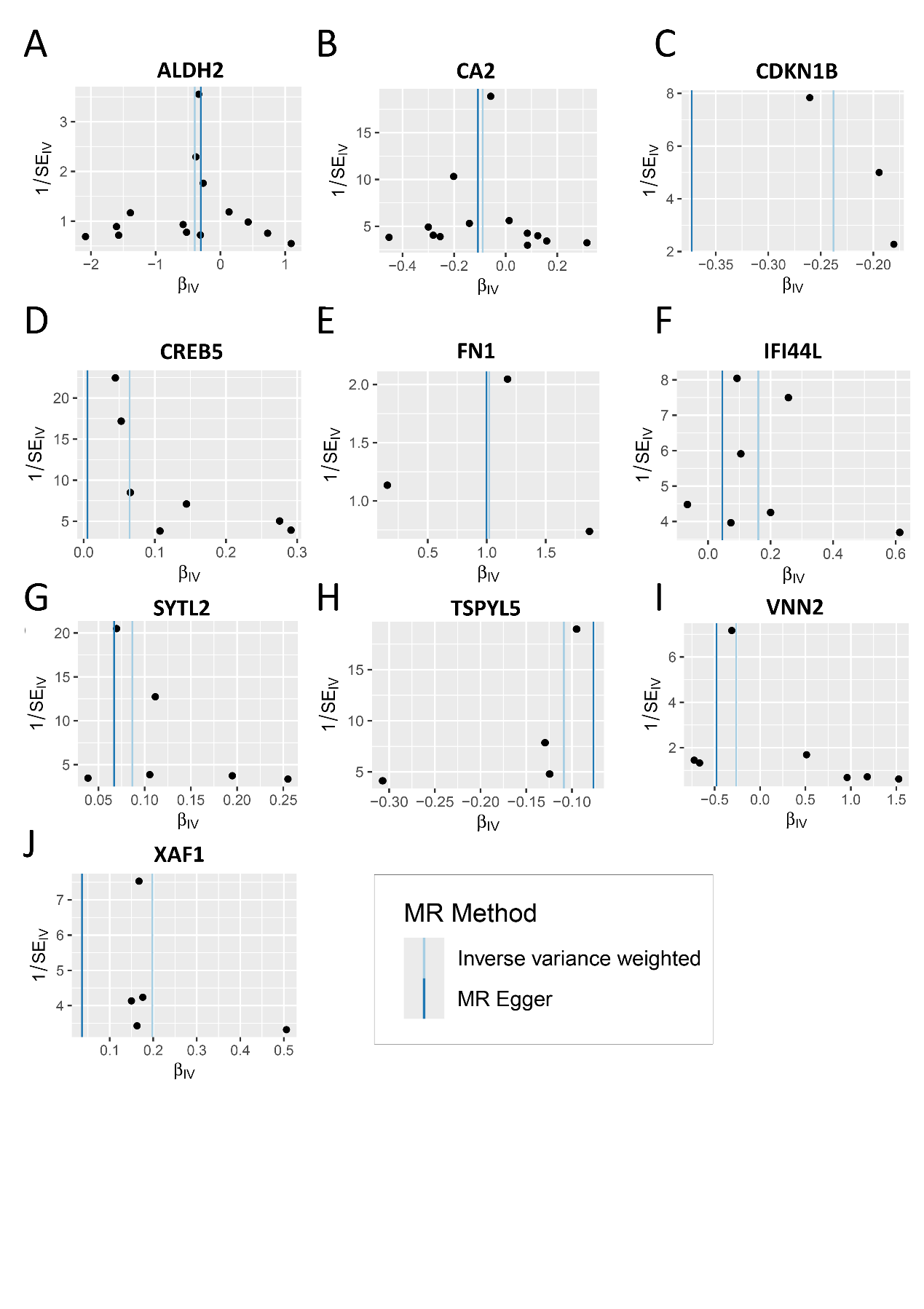


**Figure S2. Funnel plot sensitivity analysis for Mendelian randomization.**

(A-J) Funnel plots of 10 risk and protective genes assessed by inverse-variance weighted (IVW) and MR Egger methods.


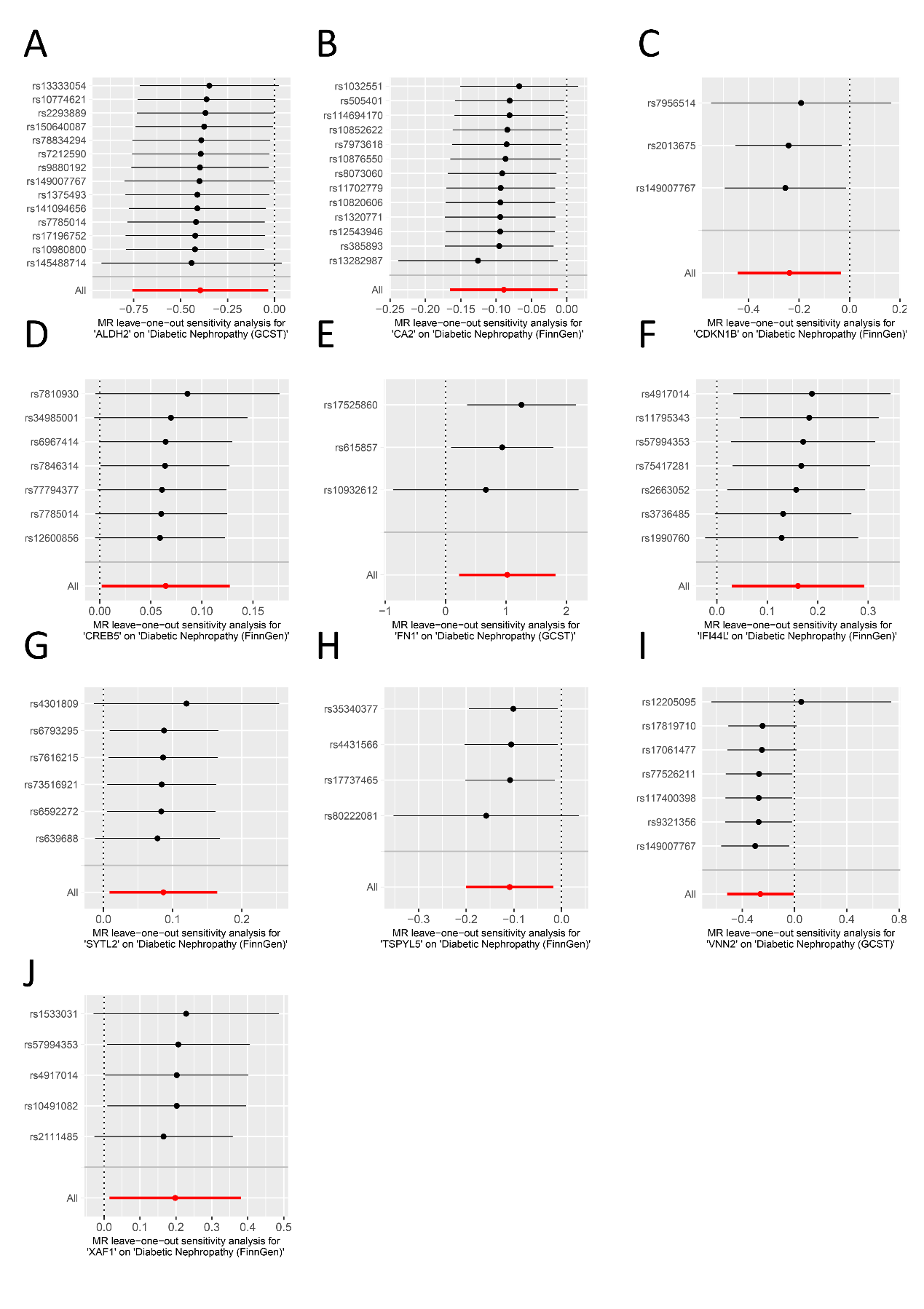


**Figure S3. Leave-one-out sensitivity analysis for Mendelian randomization.**

(A-J) Leave-one-out sensitivity tests for 10 risk and protective genes.


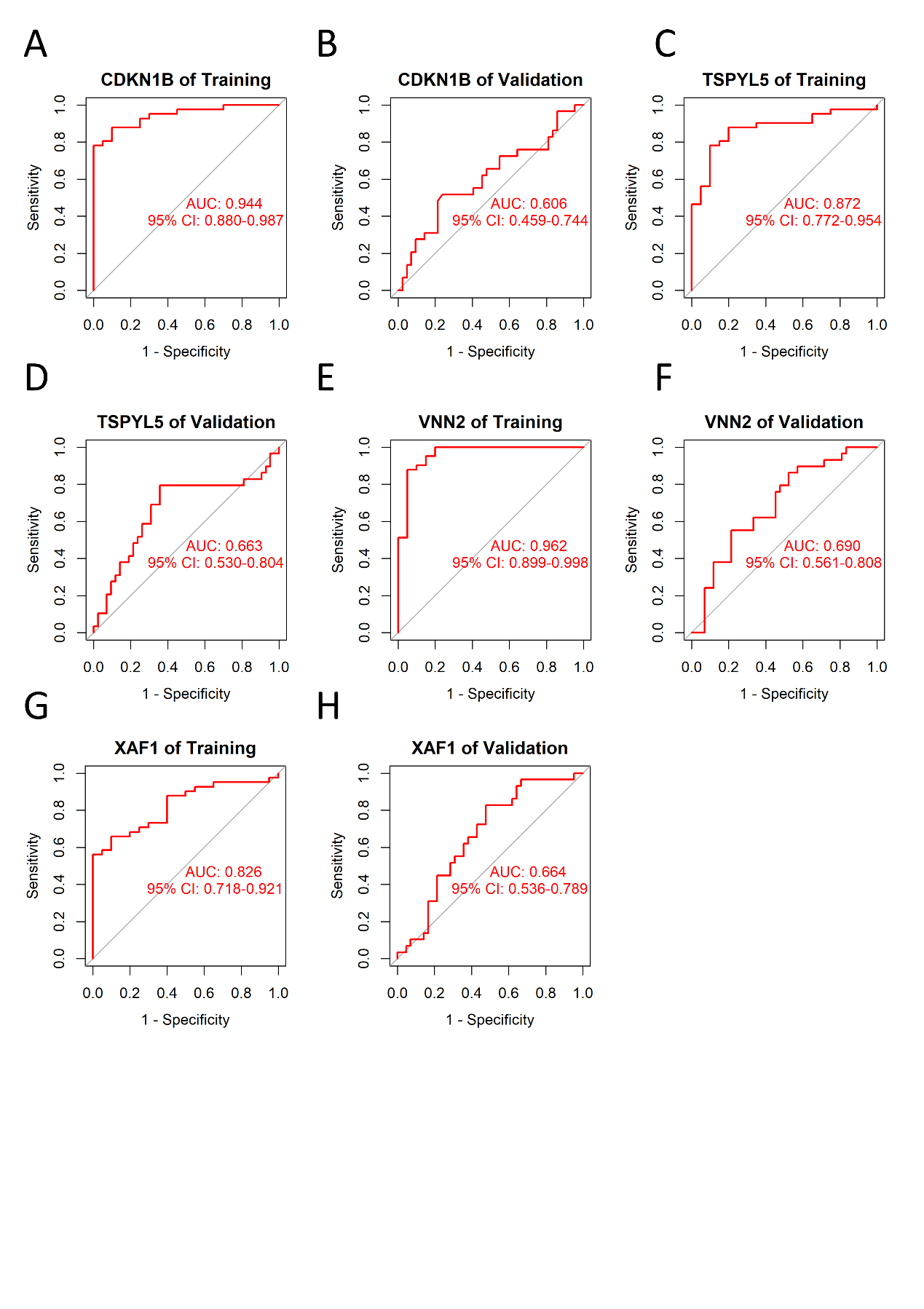


**Figure S4. Diagnostic performance of secondary candidate genes.**

(A-H) Receiver operating characteristic (ROC) curves showing AUC values of CDKN1B, TSPYL5, VNN2, and XAF1 in training and validation cohorts.


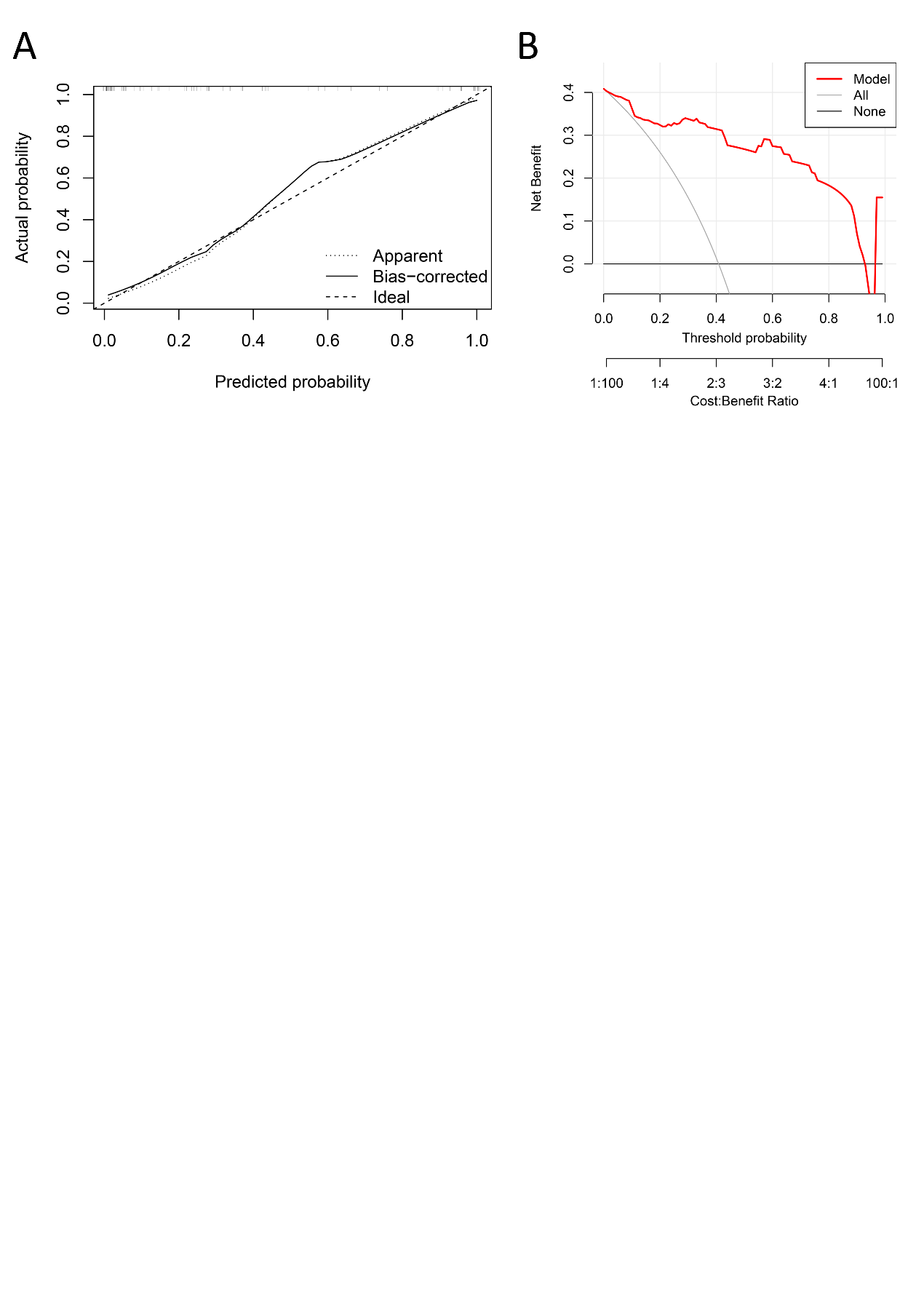


**Figure S5. Model validation using calibration and decision curve analysis.** (A) Calibration plot demonstrates good overall fit, albeit with a minor underestimation of risk for predictions between 0.4 and 0.6. (B) The decision curve indicates the model's clinical utility by offering a greater net benefit than default strategies for most threshold probabilities.


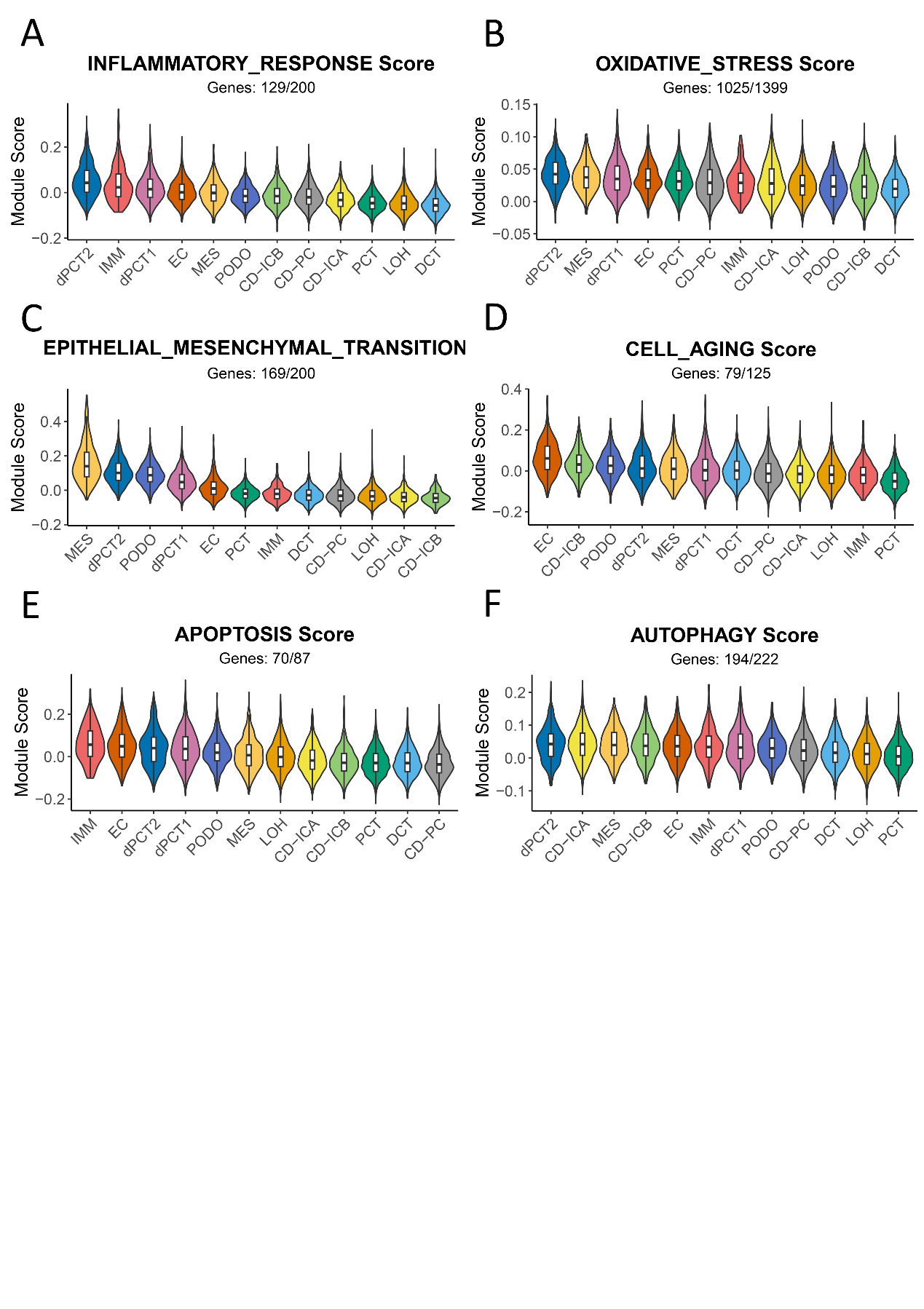


**Figure S6. Pathological pathway activation across cell subpopulations in early-stage DKD.**

(A-F) Violin plots displaying AddModuleScore enrichment scores of inflammatory response, oxidative stress, epithelial-mesenchymal transition (EMT), senescence, apoptosis, and autophagy pathways in 12 cell subpopulations from early-stage DKD.


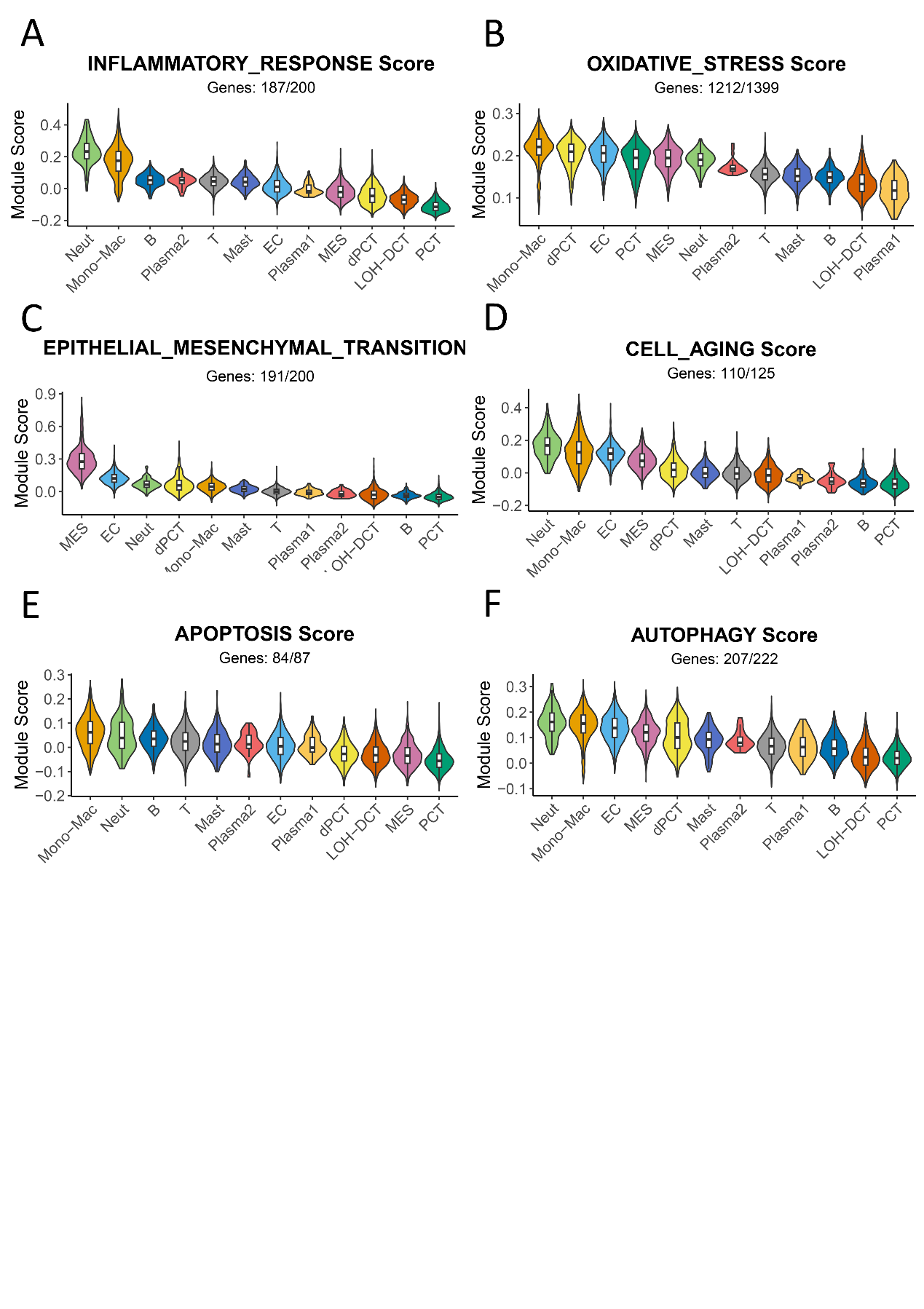


**Figure S7. Pathological pathway activation across cell subpopulations in late-stage DKD.**

(A-F) Violin plots showing AddModuleScore enrichment scores of inflammatory response, oxidative stress, EMT, senescence, apoptosis, and autophagy pathways in 12 cell subpopulations from late-stage DKD.


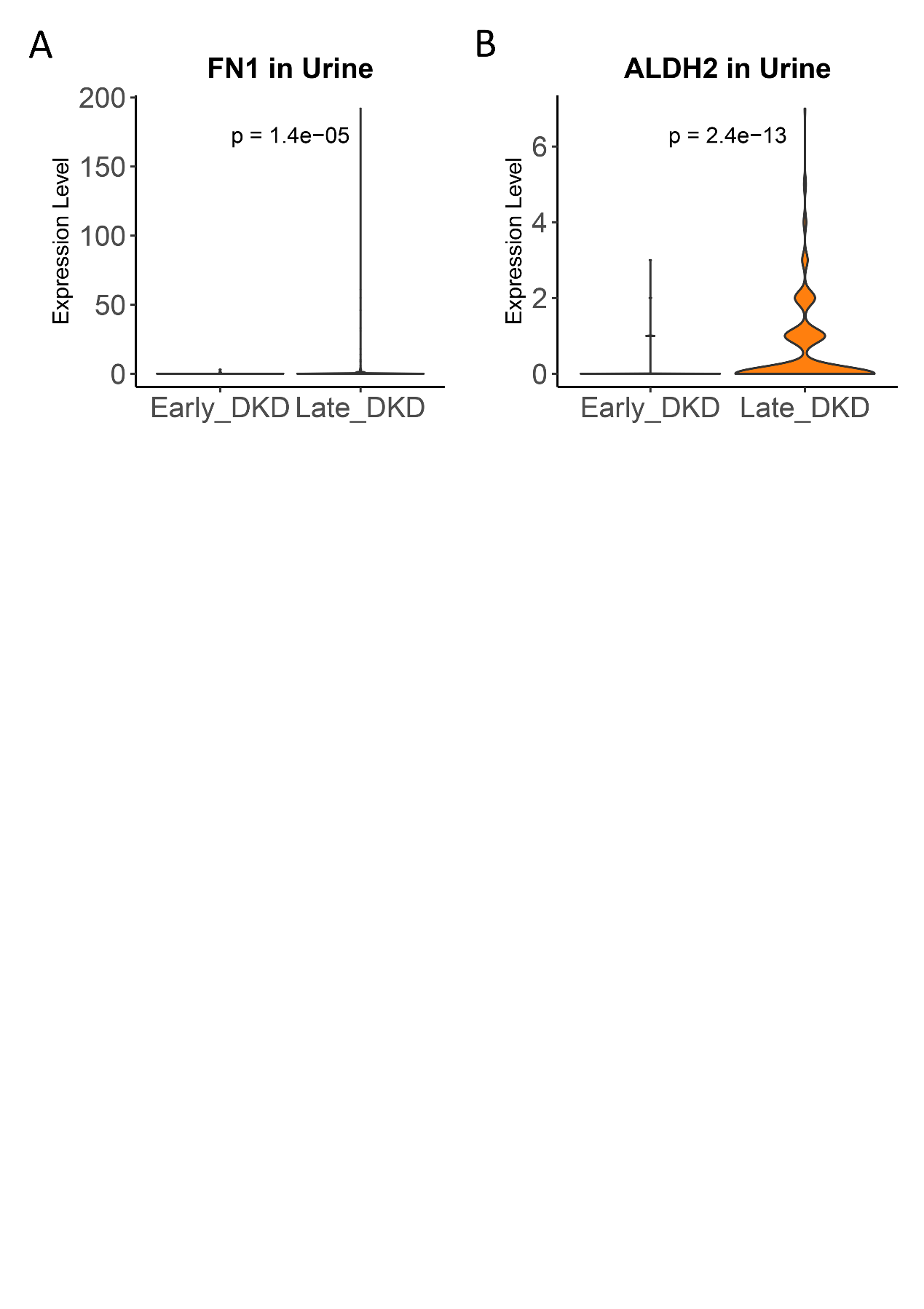


**Figure S8. Urinary single-cell exploration of ALDH2 and FN1 in DKD.**

(A-B) Violin plots comparing expression levels of ALDH2 and FN1 in urinary sediment cells between early- and late-stage DKD patients.


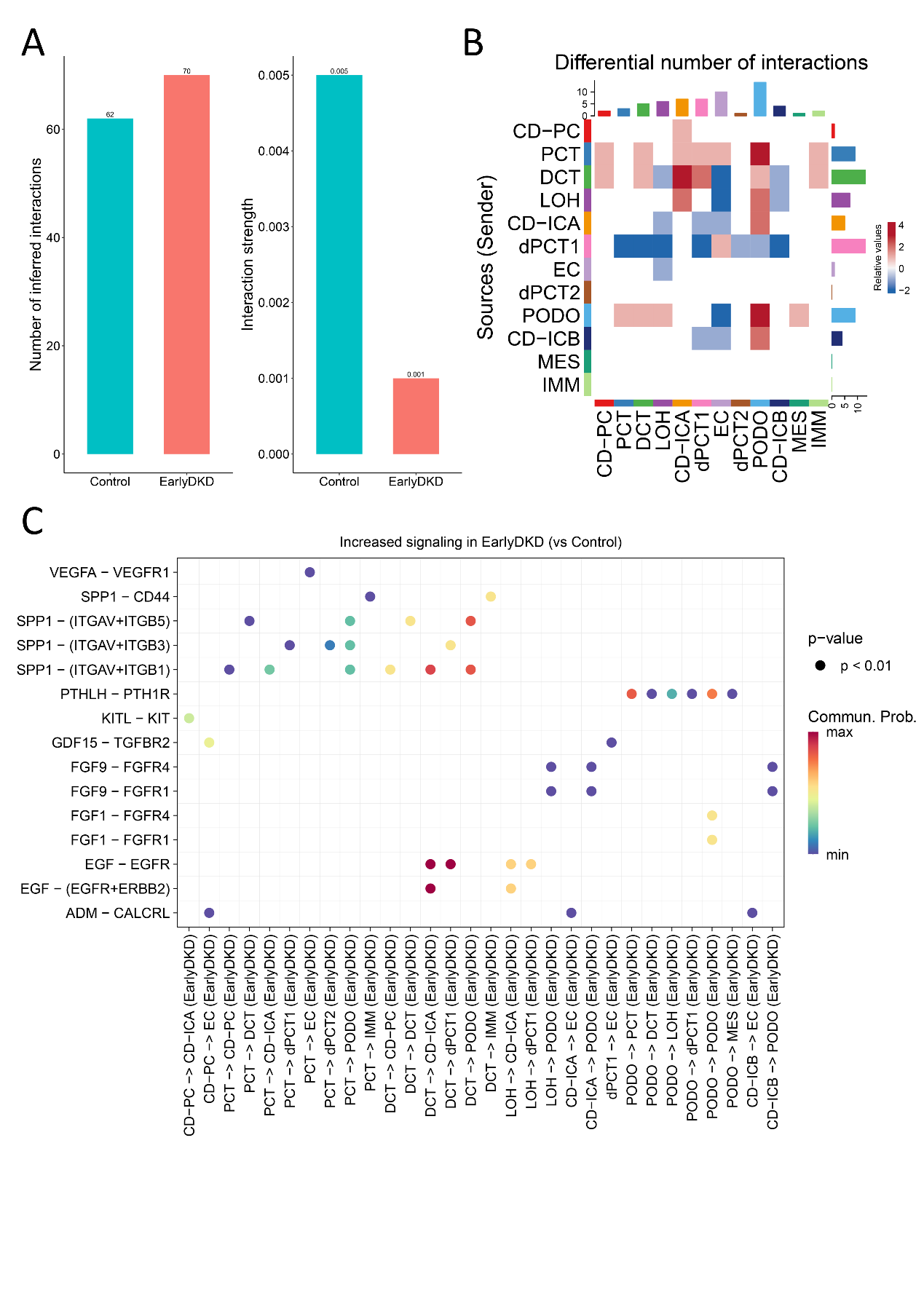


**Figure S9. Cell-cell communication network using CellChat in early-stage DKD.**

(A) Differences in the number and interaction strength of cell-cell communications between early-stage DKD and controls. (B) Heatmap showing significantly altered cell-cell interactions (red indicates increased interactions in DKD compared to controls; blue indicates decreased interactions). (C) Bubble plot showing ligand-receptor interactions between cell clusters. Redder color indicates higher communication probability; bluer color indicates lower probability.

**
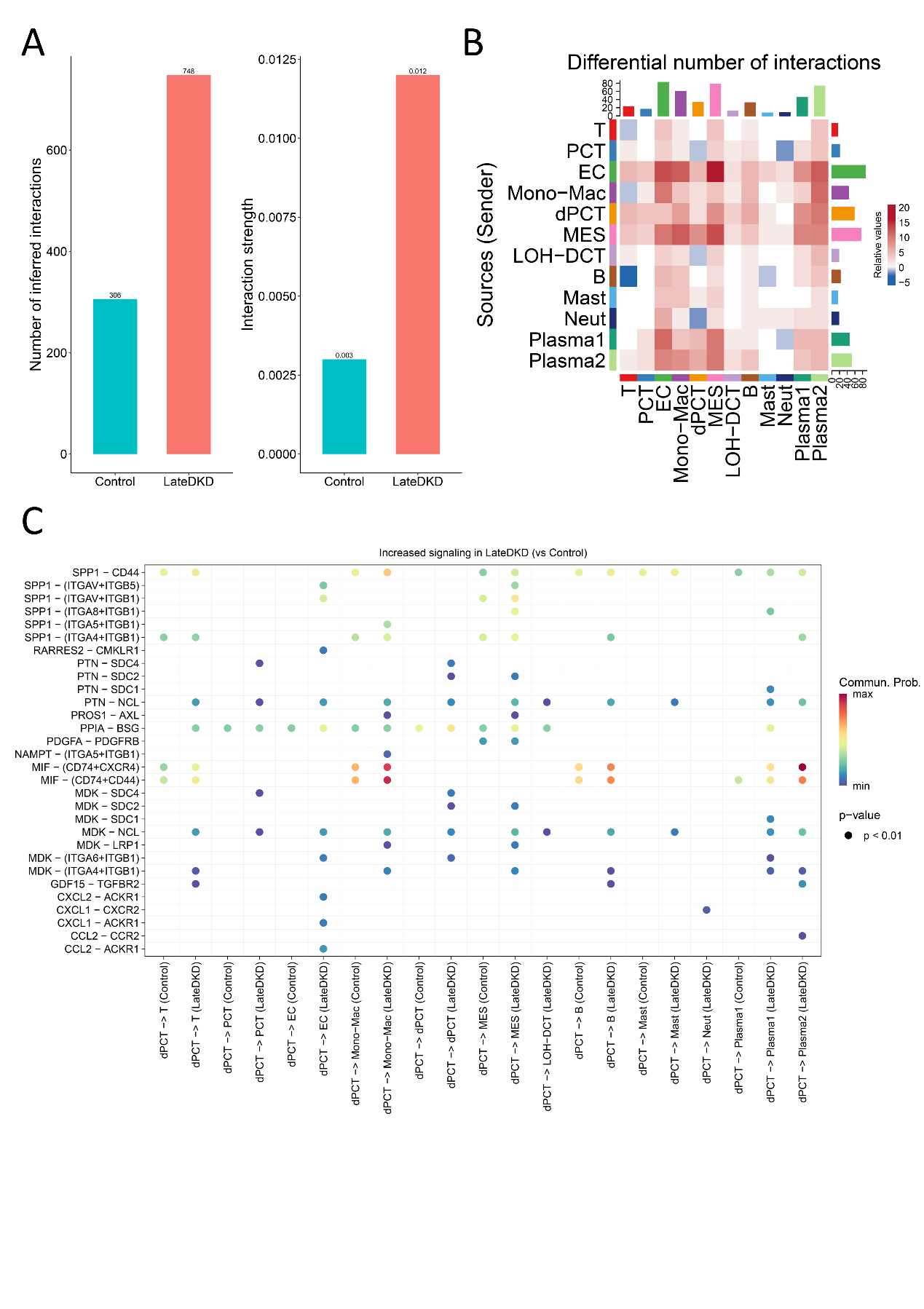
Figure S10. Cell-cell communication network using CellChat in late-stage DKD.**

(A) Changes in the quantity and intensity of cell-cell communications in late-stage DKD versus controls. (B) Heatmap showing significantly altered cell-cell interactions (red indicates increased interactions in DKD compared to controls; blue indicates decreased interactions). (C) Bubble plot of ligand-receptor interactions. Color scale: red (high probability) to blue (low probability).
